# Genome-Wide Association Study of COVID-19 Outcomes Reveals Novel Host Genetic Risk Loci in the Serbian Population

**DOI:** 10.3389/fgene.2022.911010

**Published:** 2022-07-14

**Authors:** Marko Zecevic, Nikola Kotur, Bojan Ristivojevic, Vladimir Gasic, Vesna Skodric-Trifunovic, Mihailo Stjepanovic, Goran Stevanovic, Lidija Lavadinovic, Branka Zukic, Sonja Pavlovic, Biljana Stankovic

**Affiliations:** ^1^ Laboratory for Molecular Biomedicine, Institute of Molecular Genetics and Genetic Engineering, University of Belgrade, Belgrade, Serbia; ^2^ Seven Bridges, Boston, MA, United States; ^3^ Clinic of Pulmonology, Clinical Centre of Serbia, Belgrade, Serbia; ^4^ Faculty of Medicine, University of Belgrade, Belgrade, Serbia; ^5^ Clinic for Infectious and Tropical Diseases, Clinical Centre of Serbia, Belgrade, Serbia

**Keywords:** GWAS, SARS-CoV-2, genetic markers, pneumonia, severe disease

## Abstract

Host genetics, an important contributor to the COVID-19 clinical susceptibility and severity, currently is the focus of multiple genome-wide association studies (GWAS) in populations affected by the pandemic. This is the first study from Serbia that performed a GWAS of COVID-19 outcomes to identify genetic risk markers of disease severity. A group of 128 hospitalized COVID-19 patients from the Serbian population was enrolled in the study. We conducted a GWAS comparing (1) patients with pneumonia (*n* = 80) against patients without pneumonia (*n* = 48), and (2) severe (*n* = 34) against mild disease (*n* = 48) patients, using a genotyping array followed by imputation of missing genotypes. We have detected a significant signal associated with COVID-19 related pneumonia at locus 13q21.33, with a peak residing upstream of the gene *KLHL1* (*p* = 1.91 × 10^−8^). Our study also replicated a previously reported COVID-19 risk locus at 3p21.31, identifying lead variants in *SACM1L* and *LZTFL1* genes suggestively associated with pneumonia (*p* = 7.54 × 10^−6^) and severe COVID-19 (*p* = 6.88 × 10^−7^), respectively. Suggestive association with COVID-19 pneumonia has also been observed at chromosomes 5p15.33 (*IRX, NDUFS6, MRPL36, p* = 2.81 × 10^−6^), 5q11.2 (*ESM1, p* = 6.59 × 10^−6^), and 9p23 (*TYRP1, LURAP1L*, *p* = 8.69 × 10^−6^). The genes located in or near the risk loci are expressed in neural or lung tissues, and have been previously associated with respiratory diseases such as asthma and COVID-19 or reported as differentially expressed in COVID-19 gene expression profiling studies. Our results revealed novel risk loci for pneumonia and severe COVID-19 disease which could contribute to a better understanding of the COVID-19 host genetics in different populations.

## 1 Introduction

The waning effectiveness of vaccines and new SARS-CoV-2 variants of concern indicate that the COVID-19 health threat will likely remain in the future. The course of SARS-CoV-2 infection ranges from asymptomatic and mild to severe disease, which can progress to critical illness and a lethal outcome. Besides acute disease, patients with severe symptoms are more likely to suffer from long term COVID-19 related distress. Several risk factors for the severe form of the COVID-19 disease have been identified, namely old age, male sex, non-Caucasian ethnicity, smoking, low income, obesity, and other preexisting comorbidities (COVID-19 Host Genetics Initiative, 2021; Hu and Wang, 2021; Shelton et al., 2021). Using this data has resulted in establishing prioritization strategies in the prevention and treatment of COVID-19 employed to optimally allocate limited healthcare resources to vulnerable groups.

Besides health and demographic data, variations in the human genome have also been analyzed to find genetic markers related to susceptibility to infection and severity of COVID-19. Identifying genetic markers associated with different COVID-19 outcomes would not only advance patient stratification but also reveal potential mechanisms of disease progression, point out important pathways, and contribute to drug discovery. Using the candidate gene approach, several loci showed association with severe disease or infection rate. These include *APOE4* (Kuo et al., 2020), *ACE1*, *ACE2* and *TMPRSS2* genes (Andolfo et al., 2021; Fink-Baldauf et al., 2022), *HLA* locus (Fricke-Galindo and Falfán-Valencia, 2021), and vitamin D level influencing genes (Kotur et al., 2021). Genome sequencing analysis focused on rare genetic variants implicated type I interferon immunity in COVID-19 progression (Zhang et al., 2020).

Genome-wide association study (GWAS) is a hypothesis-free approach suitable for the discovery of novel, common genetic markers. Genome-wide analyses have been focused on different COVID-19 outcomes, such as susceptibility to infection, severe disease, critical disease, and lethal outcome, as well as hospitalization rate, post-COVID-19 syndrome and response to COVID-19 vaccines (COVID-19 Host Genetics Initiative, 2021; Pairo-Castineira et al., 2021; Wu et al., 2021; Thibord et al., 2022). The results of these studies have implicated several genome loci with a COVID-19 outcome. The most replicated result so far associated variants on chromosome 3 near *LZTFL1* and *SLC6A20* genes with infection, hospitalization, and critical illness, as well as *ABO* gene on chromosome 9 correlating non-O blood type with a higher rate of infection and severe disease (Ellinghaus et al., 2020; COVID-19 Host Genetics Initiative, 2021; Pairo-Castineira et al., 2021; Shelton et al., 2021; Thibord et al., 2022).

Efforts to find genetic variants continue to include more ethnic groups and COVID-19 outcomes. In this study, GWAS was performed on COVID-19 patients of Serbian origin in order to identify genetic markers of different COVID-19 phenotypes.

## 2 Materials and Methods

### 2.1 Subjects and Genotyping

A total of 216 participants were recruited for the study. Participants were confirmed positive for COVID-19 according to local clinical testing (SARS-CoV-2 RNA RT-PCR) in tertiary healthcare institutions in Belgrade, Serbia, between April and June of 2020. Whole blood samples were taken from the participants and further used for DNA extraction. All subjects were genotyped using the Illumina Infinium Global Screening Array v.3.0 + Multi-Disease BeadChip (GSAMD-24v3-0-EA), a high-density array that covers over 700,000 variants. The obtained data was used to build a population-specific cluster file.

From the total study group, we selected 128 adult patients that satisfied all criteria needed for the phenotypic classification used in association analysis. Precisely, patients were divided into three groups according to the National Institutes of Health (NIH) Definition of COVID-19 Disease Severity [COVID-19 Treatment Guidelines Panel. Coronavirus Disease 2019 (COVID-19) Treatment Guidelines. National Institutes of Health. Available at https://www.covid19treatmentguidelines.nih.gov/. Accessed 01 September 2021]: mild—patients with COVID-19 related symptoms without pneumonia; moderate—patients with evidence of pneumonia based on imaging showing up to 50% of lung involvement and with oxygen saturation ≥94% on room air; and severe—patients with pneumonia with >50% of lung involvement on imaging or had blood oxygen saturation level <94% on room air or required supportive oxygen therapy.

Patients included in the study were not vaccinated against COVID-19 at the time of diagnosis. Informed consent was obtained from each participant or their parents/legal guardians. This study was approved by the Ethics Committee of the Institute of Molecular Genetics and Genetic Engineering University of Belgrade (approval for sample collection and biobank formation O-EO-016/2020, 05 May 2020; approval for the genetic study O-EO-016/2020/1, 03 September 2020).

### 2.2 Data Analysis

#### 2.2.1 Data Preprocessing, Variant Calling and Imputation

This study analyzed samples from the Serbian population using the custom GSAMD panel which required generating a population-specific cluster file. Initial genotype calling from GSAMD intensity data (IDAT) files and quality control (QC) analysis before cluster file generation was performed using GenomeStudio v.2.0 software with GSAMD-24v3-0-EA_20034606_A1.bpm manifest file based on the human genome assembly hg19. Details regarding cluster file generation and initial QC analysis are provided in the Supplementary Methods.

The cluster file and lists of samples and variants that passed QC filters were further used as inputs for the GWAS analysis pipeline that we have set up. The pipeline encompasses variant calling, phasing, and imputation, as well as QC analysis (described in detail in the Supplementary Methods). After the imputation and prior to the genome-wide association analysis, our dataset contained 12,001,939 variants.

#### 2.2.2 Genome-Wide Association Study

The GWAS analysis pipeline was based on the GENESIS v2.10.0 (Gogarten et al., 2019) R/Bioconductor package and the various methods it implements. It has provided us with a mixed model framework that accounts for genetic relatedness and allows for the inclusion of different risk factors as covariates.

We fitted a generalized linear mixed model (GLMM) containing both fixed effects (independent covariates) and random effect which models the genetic correlation between the individuals (kinship matrix), under the null hypothesis of no genotype effects. Previous studies have identified several independent risk factors, including age and male sex (Hu and Wang, 2021). In our model formula, we have included age and sex as well as an interaction term (age*sex) between the two.

The fitted null model was then used for single variant association testing and score tests were performed for all variants with minor allele count (MAC) ≥ 10 individually. We have chosen to apply the saddle point approximation (SPA) to the score test statistic to estimate the null distribution. The two significance thresholds utilized were those of genome-wide significance (*p* < 5 × 10^−8^)—representing a Bonferroni-corrected 5% family-wise error rate threshold for the estimated effective number of 1000000 independent common genetic variants given the linkage disequilibrium structure of the human genome (Uffelmann et al., 2021); and suggestive association (*p* < 1 × 10^−5^)—a less stringent threshold intended for the identification of SNPs that should be considered in follow-up studies.

Detailed information on the genome-wide association analysis methods that we applied is provided in Supplementary Methods.

#### 2.2.3 Post-GWAS Analysis

Bayesian fine-mapping from GWAS summary statistics of the loci reaching genome-wide significance was performed using the SuSiE method implemented in the R package susieR (Zou et al., 2021) to determine the posterior inclusion probability (PIP) for each variant being causal as well as to determine a credible set, which is the smallest set of variants that contains all the causal variants with a probability ≥0.95%.

LD clumping and variant annotation were performed using the FUMA v1.3.7 web application (Watanabe et al., 2017). Variant annotation for each locus was made on independent significant variants and all the variants in LD with them that are less than 250 kb away, and with a score test *p*-value < 0.05.

Next, we conducted an exploratory analysis of the annotated variants (independent significant variants and their LD proxies) in order to identify causal variants that have a deleterious gene effect or effects on gene expression, by employing different genome browsers (Ensembl (Howe et al., 2021), The University of California Santa Cruz, UCSC (Lee et al., 2022)) and web-based tools and databases such as those from the LDlink v5.2 suite: LDproxy and LDexpress (Machiela and Chanock, 2015; Lin et al., 2021)—linked to Regulome and GTEx databases, HaploReg v4.1 (Ward and Kellis, 2012)—linked to Roadmap Epigenomics and ENCODE projects as well as different eQTLs studies data, GeneAtlas (Canela-Xandri et al., 2018) and FUMA which uses information from 18 different repositories (Watanabe et al., 2017). For eQTL mapping, the window size was defined as 1 Mb upstream and downstream from the transcription start site.

Through the FUMA web app, by utilizing the Multi-marker Analysis of GenoMic Annotation (MAGMA) (de Leeuw et al., 2015) method, genes and gene-sets associated with COVID-19 severity were analyzed, based on the signals identified at GWAS loci. To perform functional analysis, first, gene-mapping had to be performed, selecting genes located up to 10 kb upstream or downstream from variants that are functionally annotated (i.e., having a functional consequence on gene expression) and showing a suggestive association with the disease severity (*p* < 1 × 10^−5^).

#### 2.2.4 Comparative Population Analysis

We investigated effect allele frequencies of GWAS identified risk loci in world-wide populations. The effect allele frequencies (AF) were extracted from the 1,000 Genome Project (1kGP) including European populations (Italy, Spain, Finland, Great Britain and USA with European ancestry) as well as Eastern Asians, South Asians, African and Ad-Mixed American (Central and South American populations) (Auton et al., 2015). We examined the level of genetic variability among populations at each risk loci by looking at the maximal global differences in allele frequencies (delta AF, dAF) calculated by subtracting the minimum from the maximum effect allele frequency across analyzed population groups.

## 3 Results

### 3.1 Study Group

A group of 216 participants diagnosed with COVID-19 (102 males, 114 females) was included in the genotyping part of the study in order to create a cluster file specific for the Serbian population. Of the total genotyping group, 16 subjects (8 males and 8 females) were excluded due to the low call rate (<0.95) during initial QC, leaving 200 samples for the cluster file generation.

For the association analysis of the COVID-19 severity, a subgroup of 128 hospitalized COVID-19 patients was selected and classified into three phenotypic groups: mild (*n* = 48), moderate (*n* = 46), and severe (*n* = 34). Demographic and clinical data for the study group are summarized in Table 1. In addition, the ancestry of our study group was defined using the principal component analysis: almost all of our participants clustered with the European population (98.4%), and only 2 patients clustered between European and Ad Mixed American populations (Supplementary Figure S1).

**TABLE 1:** COVID-19 patients’ demographic and clinical data.

	Mild	Moderate	Severe	*p*
N (%)	48 (37.5)	46 (35.9)	34 (26.6)	
Age, median [IQR]	39.0 [29.0–49.0]	45.5 [36.0–61.0]	61.0 [50.0–68.5]	<0.0001
Gender, male n (%)	12 (25.0)	24 (52.2)	22 (64.7)	0.0009
Obesity, n/available (%)	6/42 (14.3)	9/36 (25.0)	9/27 (33.3)	0.17
Diabetes, n/available (%)	1/48 (2.1)	4/46 (8.7)	6/33 (18.2)	0.03
Hypertension, n/available (%)	8/48 (16.7)	14/46 (30.4)	18/33 (54.5)	0.0015
ACE inhibitors, n/available (%)	5/48 (10.4)	7/43 (16.3)	13/30 (43.3)	0.002
% SatO2, median [IQR]	98 [98–99]	98 [97–99]	90 [85–96]	<0.0001
CRP, median [IQR]	1.1 [0.5–5.4]	10.3 [3.0–33.3]	128.0 [60.6–200.0]	<0.0001
Febrile, n/available (%)	15/47 (31.9)	34/46 (73.9)	30/33 (90.9)	<0.0001
Lymphopenia, (≤1 × 10^9^/L), n/available (%)	12/46 (26.1)	23/46 (50.0)	24/32 (75.0)	0.0001
Thrombocytopenia, (<150,000/mm3), n/available (%)	8/45 (17.8)	8/46 (17.4)	17/32 (53.1)	0.0005

Each count was presented along with the total available number of observations for that category (n/available). Differences between the groups were tested using the Kruskal–Wallis test for continuous data, Chi-square, or Fisher exact test for discrete data. IQR - interquartile range, SatO2 - blood oxygen saturation, CRP - C-reactive protein.

In the COVID-19 study group, 12 patients (9.4%) required supportive oxygen therapy and 4 (3.1%) had COVID-19 related death outcomes. The age of patients significantly varied between the groups, being the highest in the severe group (*p* < 0.0001). Gender distribution was different among the mild, moderate, and severe groups, including 25%, 52.2%, and 64.7% of male patients, respectively (*p* = 0.0009). Patients with severe disease more frequently suffered from diabetes and hypertension (*p* = 0.03, *p* = 0.0015, respectively). CRP level was prominently higher in the severe group and those patients had a higher percentage of lymphopenia (75%) and thrombocytopenia (53.1%) events compared to patients with moderate and mild disease (*p* < 0.0001, *p* = 0.0001 and *p* = 0.0005, respectively).

### 3.2 Severity Loci Identified by the Genome-Wide Analysis

To assess the genetic component of risk for different COVID-19 outcomes in the Serbian population, we have performed a genome-wide association analysis, testing for genetic variant allele frequency differences between groups of patients classified as either mild (*n* = 48), moderate (*n* = 46) or severe (*n* = 34).

**FIGURE 1:**
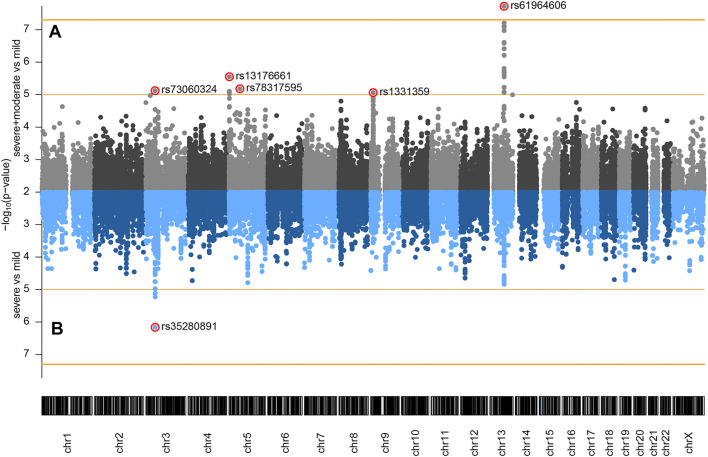
Genome-wide association of COVID-19 outcomes in Serbian population: **(A)** severe and moderate (*n* = 80) versus mild (*n* = 48); **(B)** severe (*n* = 34) versus mild (*n* = 48) comparisons. Genomic coordinates of analyzed SNPs are displayed along the *x*-axis and the observed statistical significance of association with the disease outcome on the negative log_10_ scale is displayed on the *y*-axis. SNPs with *p* < 0.01 are not shown on the plot. The two horizontal orange lines represent the two significance thresholds used: genome-wide significance (*p* < 5 × 10^−8^, y ≈ 7.3) and suggestive association (*p* < 1 × 10^−5^, y = 5).

The following comparisons have been made: 1) severe and moderate versus mild, 2) severe versus mild, and 3) severe versus moderate and mild. Genome-wide association analysis of severe and moderate versus mild, which actually compares patients with pneumonia against patients with no pneumonia diagnosed, identified a significant association signal at locus 13q21.33 (Figure 1A). In the two other comparisons, we did not observe signals reaching genome-wide significance (*p* < 5 × 10^−8^), but several signals showed a suggestive association with disease severity (*p* < 1 × 10^−5^). Severe versus mild comparison gave a stronger signal at the already recognized risk locus at chromosome 3 (Figure 1B) than severe versus moderate and mild (Supplementary Figure S2), probably due to severe and moderate categories not being as distinctly separated as moderate and mild. Hence, we focused our efforts on the first two genotypic-phenotypic comparisons: testing for differences in the allele frequency of genetic variants in (1) patients with pneumonia (severe and moderate patients grouped together) versus those without pneumonia (mild disease group) and (2) in patients diagnosed with pneumonia with above 50% lung involvement or blood oxygen level below 94% (severe disease group) versus patients without pneumonia (mild disease group). In both genotypic-phenotypic comparisons, patients who had a mild disease (without pneumonia) served as a reference or control group in statistical analyses.

#### 3.2.1 Genome-Wide Association of COVID-19 Related Pneumonia (Severe and Moderate Versus Mild Disease)

We have performed GWAS comparing 80 patients from the severe and moderate category versus 48 from the mild disease category. The total number of SNPs analyzed by fitting a logistic mixed effect model adjusted for age, sex, and genetic relatedness was 7496155. Genomic inflation factor lambda (λ) was 0.9959 and the quantile-quantile (QQ) plot is shown in Supplementary Figure S3.

**FIGURE 2:**
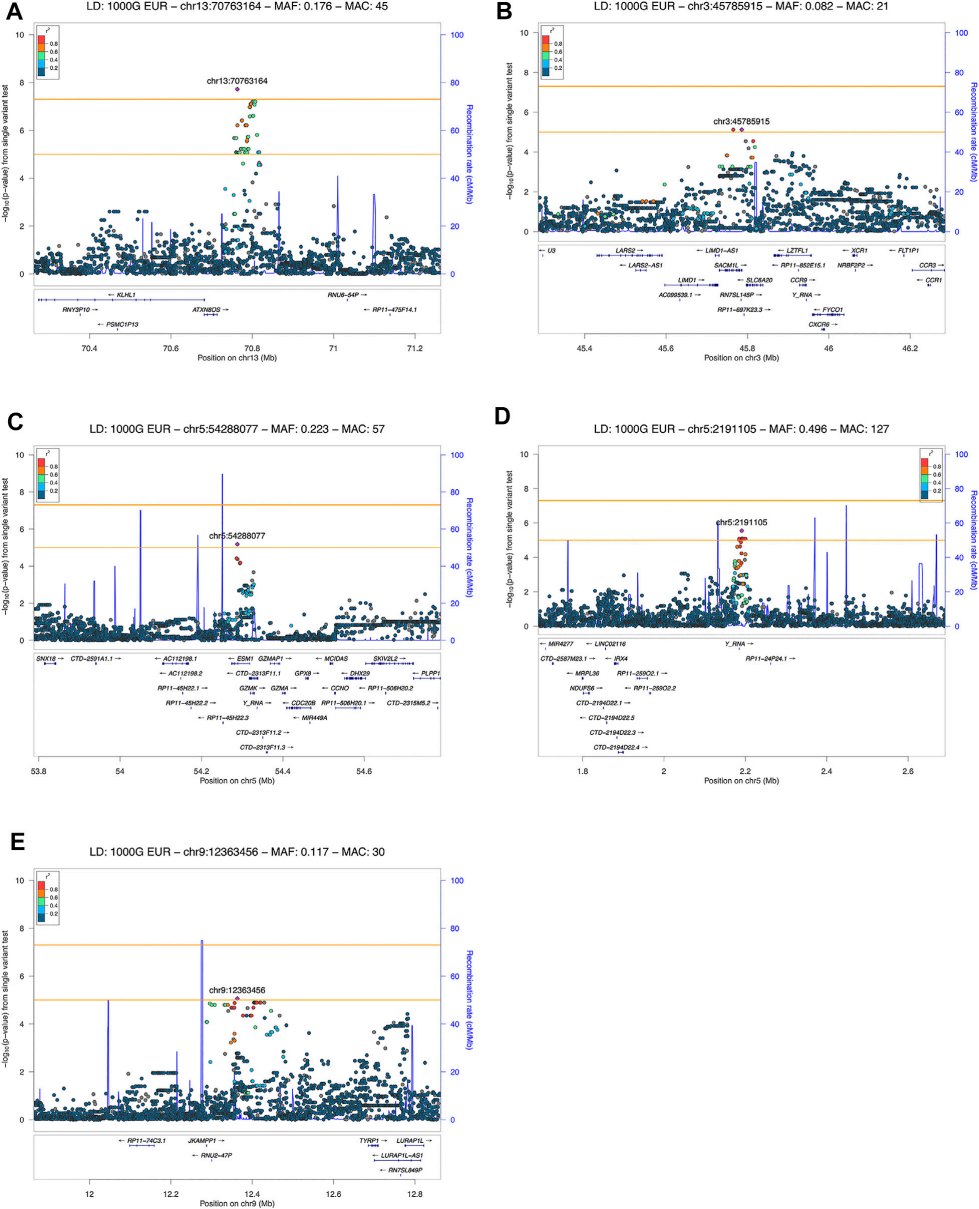
LocusZoom plot showing the regional association of GWAS variant enriched loci with COVID-19 severity (severe and moderate versus mild patient group comparison). Variants in linkage disequilibrium with the lead variant are shown in color gradient indicating r^2^ levels (hg19, 1KGP, Phase 3—November 2014, EUR): **(A)** 13q21.33 with rs61964606 as the lead variant; **(B)** 3p21.31 with rs73060324 as the lead variant; **(C)** 5p15.33 with rs13176661 as the lead variant; **(D)** 5q11.2 with rs78317595 as the lead variant; **(E)** 9p23 with rs1331359 as the lead variant.

We detected 5 risk loci, namely 13q21.33, 3p21.31, 5p15.33, 5q11.2, and 9p23, showing at least a suggestive association with COVID-19 related pneumonia (Table 2). The imputation quality of the associated independent variants was high (allelic r^2^ > 0.82). Regional association plots for the identified risk loci are shown in Figure 2. The annotated independent significant variants together with the variants that they are in linkage disequilibrium with (r^2^ > 0.6) are presented in Supplementary Table S2.

**TABLE 2:** Lead variants: severe and moderate versus mild disease.

Lead Variant	Cytoband	Position (hg19)	*p*-value	Nearest gene(s)	Location	Ref	Alt (effect allele)	Alt allele frequency	Effect size (beta)	OR	95% CI	r^2^
rs61964606	13q21.33	70763164	1.91 × 10^−8^	*KLHL1, ATXN8, ATXN80S*	intergenic	A	G	0.824	2.314	10.115	4.458–22.949	0.903
rs73060324	3p21.31	45785915	7.54 × 10^−6^	*SACM1L*	3'-UTR	T	G	0.082	−2.473	0.084	0.028–0.257	0.984
rs13176661	5p15.33	2191105	2.81 × 10^−6^	*IRX4, NDUFS6, MRPL36*	intergenic	G	A	0.504	−1.391	0.249	0.138–0.449	0.974
rs78317595	5q11.2	54288077	6.59 × 10^−6^	*ESM1*	intronic	T	C	0.223	−1.727	0.117	0.083–0.379	0.956
rs1331359	9p23	12363456	8.69 × 10^−6^	*TYRP1, LURAP1L*	intergenic	G	A	0.117	−2.309	0.099	0.035–0.279	0.999

Lead variants representing 5 genomic loci and showing at least a suggestive association (*p* < 1 × 10^−5^) with the disease severity when comparing patients with pneumonia (severe and moderate disease groups) versus those without pneumonia (mild disease group). Summary statistics such as *p*-value, alternative allele frequency in our cohort, effect size estimate for each additional copy of the alternative allele, and odds-ratio (OR) are shown as well the imputation quality metric (r^2^). Ref – reference allele, Alt – alternative allele, CI – confidence interval.

Out of the 5 loci, only 13q21.33 achieved genome-wide significance with rs61964606 as its lead variant. The major G allele of this variant conferred increased risk for COVID-19 related pneumonia (OR = 10.115, 95% CI: 4.458–22.949, *p* = 1.91 × 10^−8^). The rs61964606 variant is located in the intergenic region upstream of the *KLHL1* gene (∼80 kb), surrounded by regulatory sequences—enhancers, transcriptional and CCCTC-binding factor (CTCF) sites. In the proximity of the lead variant, we detected transcriptional factors JUND and FOXA1 binding sites according to Open Regulatory Annotation database (ORegAnno) which is incorporated into the UCSC genome browser (Lesurf et al., 2016). Linkage disequilibrium (0.6 > r^2^ > 0.5) was found between our lead variant and several eQTL variants associated with *KLHL1* gene expression in brain tissue (rs7339068, rs9542235, rs7339309, all three *p* = 2.2 × 10^–7^, based on PsychENCODE database (Wang et al., 2018)).

Fine-mapping from summary statistics enabled us to examine the posterior inclusion probability (PIP) for each variant on the genomic risk locus on chromosome 13 (from chr13:70755332 to chr13:70819769). The 95% credible variant set is shown in Supplementary Table S1.

Our genome-wide analysis detected a suggestive signal in replicated COVID-19 risk locus at chromosome 3p21.31, in the 3′-UTR of the *SACM1L* gene (OR = 0.084, 95% CI: 0.028–0.257, *p* = 7.54 × 10^−6^). Detected lead *SACM1L* rs73060324 variant highly correlates with rs17279437 missense variant (r^2^ = 0.88) located in the *SLC6A20* gene, a potential causative candidate which has been previously associated with COVID-19. Predicted pathogenicity of rs17279437 was high (CADD score = 25.4, Polyphen = 1). Our results showed that allele A of this variant was associated with a lower risk of pneumonia in COVID-19 patients.

Regarding other suggestive loci, the signal at 5q11.2 was located in the intron of the *ESM1* gene. Functional annotation showed that lead variant rs78317595 was in LD with eQTL rs10076939 (r^2^ = 0.74) associated with *ESM1* gene expression in human blood vessel tissue (*p* = 10^−4^, based on GTEx v8). The remaining two signals were found in intergenic regions: 5p15.33 was proximal to *IRX4, NDUFS6,* and *MRPL36,* while 9p23 was close to *TYRP1 and LURAP1L* genes (both at ∼ 300–400 kb distance from nearest genes). Intergenic signal near *IRX4, NDUFS6,* and *MRPL36* was located in the region highly enriched with regulatory sequences. According to Roadmap Epigenomics data, the rs13172851 variant (high LD with lead variant rs13176661, r^2^ = 0.88), lies in the enhancer histone marks (H3K4me1_Enh and H3K27ac_Enh) active in various tissues, including lungs.

#### 3.2.2 Genome-Wide Association of Severe COVID-19 (Severe Versus Mild Disease)

The results of genome-wide association analysis of severe (*n* = 34) versus mild (*n* = 48) Serbian COVID-19 patients include only a single locus—3p21.31, that reached the suggestive, but not the genome-wide significance threshold (*p* = 6.88 × 10^−7^) (Table 3). The model type and the covariates chosen were the same as in the comparison that was described in the previous section. The total number of variants analyzed was 6,695,505; genomic inflation factor lambda (*λ*) was 0.9944 and the quantile-quantile (QQ) plot is shown in Supplementary Figure S4.

**TABLE 3:** Lead variants: severe versus mild COVID-19.

Lead Variant	Cytoband	Position (hg19)	*p*-value	Nearest Gene	Location	Ref	Alt (effect allele)	Alt Allele Frequency	Effect Size (beta)	OR	95% CI	r^2^
rs35280891	3p21.31	45951647	6.88 × 10^−7^	*LZTFL1*	intronic	G	A	0.116	2.988	19.846	5.728–68.761	0.762

The lead variant in the 3p21.31 locus showed a suggestive association (p < 1 × 10^−5^) with the disease severity when comparing patients from the severe versus those from the mild disease group. Summary statistics such as *p*-value, alternative allele frequency in our cohort, effect size estimate for each additional copy of the alternative allele, and odds-ratio (OR) are shown as well the imputation quality metric (r^2^). Ref – reference allele, Alt –alternative allele, CI – confidence interval

**FIGURE 3:**
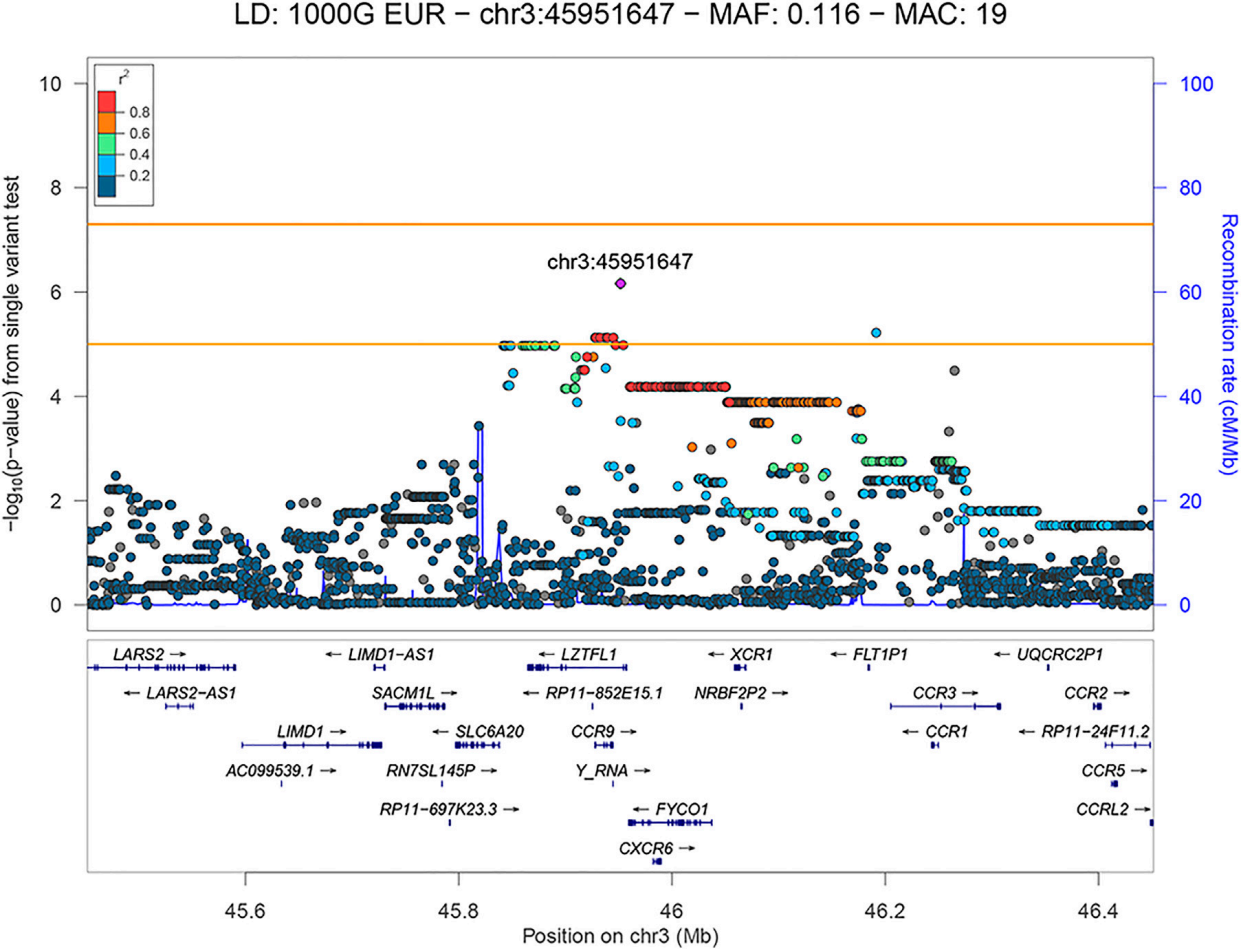
LocusZoom plot showing the regional association of 3p21.31 with COVID-19 severity (severe versus mild patient group comparison), with rs35280891 as the lead variant. Variants in linkage disequilibrium with rs35280891 are shown in color gradient indicating r^2^ levels (hg19, 1KGP, Phase 3—November 2014, EUR).

We have observed a total of 10 variants in the 3p21.31 locus that showed a suggestive association, all of which are shown in the regional association plot in Figure 3. The variants were found to be physically close together (within a ∼260 kb region) and in LD with each other (r^2^ ≥ 0.725; based on the 1kGP Phase 3 data—EU super-population) with the lead variant being rs35280891 (OR = 19.846, 95% CI: 5.728–68.761, *p* = 6.88 × 10^−7^). The imputation quality of the associated variants was high (r^2^ > 0.5844). The annotated independent significant variants together with the variants they are in linkage disequilibrium with (r^2^ > 0.6) are presented in Supplementary Table S3.

The lead variant is in a high LD with three missense variants (r^2^ > 0.84) located in the *FYCO1* gene: rs13079478, rs13059238 and rs33910087 (all three: OR = 11.107, 95% CI: 3.262–37.817, *p* = 6.57 × 10^−5^). Variants rs13079478 and rs13059238 are located at the same codon, so three missense variants affect two amino acids of the resulting polypeptide. According to GeneAtlas, *FYCO1* variant rs33910087 is a highly significant modifier of monocyte percentage (*p* = 3.85 × 10^−46^), and based on GTEx v8, this variant is also an eQTL for several protein-coding genes previously associated with COVID-19 (*CXCR6*, *FYCO1*, *SLC6A20*, *CCR1*, *LZTFL1*). Also, independent significant variant rs192311430, located in the intron of the *LZTFL1* gene, is associated with *CCR3* and *FLT1P1* gene expression in whole blood (*p* = 3 × 10^−5^ and *p* = 2 × 10^−5^, respectively), as well as with *CCR6* gene expression in neural tissue (*p* = 2 × 10^−4^), based on GTEx v8.

### 3.3 Functional Analysis

In both comparisons (severe and moderate versus mild, and severe versus mild), the gene set enrichment analysis of genes located up to 10 kb upstream or downstream from a candidate variant showed significant enrichment of chemokine related pathways (*REACTOME_CHEMOKINE_RECEPTORS_BIND_CHEMOKINES*, FDR adjusted *p*-value = 6.32 × 10^−10^, 8 out of 48 genes from this gene set have been found near our candidate variants: *CCR9, CXCR6, XCR1, CCR3, CCR1, CCR2, CCR5, CCRL2*).

### 3.4 Comparative Population Analysis

Frequencies of the lead variants in the worldwide populations have been extracted from the 1kGP (Supplementary Table S4) and visualized along with the Serbian population in Supplementary Figure S5.

Examination of entries in the 1kGP database for risk loci showed that the frequency of effect alleles among worldwide populations varied highly (highest dAF, which is a difference between maximal and minimal effect allele frequency) for rs13176661, ranging from 91% in African to 56% in European populations, being the lowest in Serbian population 50%, and rs1331359, ranging from 95% in African to 46% in East Asian populations.

### 3.5 Validation of Lead Variants in Publicly Available COVID-19 Genome-Wide Association Studies

Since another cohort of COVID-19 patients from the Serbian population was not available, we relied on publicly available GWAS datasets of COVID-19 patients for validation of obtained associations. In order to validate lead variants as novel risk markers of COVID-19 severity, we employed UK biobank data of COVID-19 positive patients of European origin. Two phenotypes similar to the ones we analyzed were selected: severe versus non-severe COVID-19 positive and hospitalized positive versus non-hospitalized positive (https://grasp.nhlbi.nih.gov/Covid19GWASResults.aspx) (Thibord et al., 2022). We considered an association validated if at least a nominally significant result (*p* < 0.05) in the UK biobank was noted in the same direction of association. The results of this analysis showed that:

1) Variant rs35280891 at 3p21.31 suggestively significant in our GWAS (*p* = 6.88 × 10^−7^) was nominally significant in both UK biobank comparisons—severe versus non-severe and hospitalized positive versus non-hospitalized positive phenotypes (*p* = 2.22 × 10^−4^ and *p* = 7.57 × 10^−7^, respectively).
2) Variant rs73060324 at 3p21.31 suggestively significant in our GWAS (*p* = 7.54 × 10^−6^) showed a statistical trend related to UK biobank hospitalized positive versus non-hospitalized positive phenotype (*p* = 0.0748).
3) Variant rs78317595 at 5q11.2 suggestively significant in our GWAS (p = 6.59 × 10^−6^) was nominally significant in the UK biobank severe versus non-severe comparison (*p* = 0.042). However, the odds ratio for the effect allele C was below 1 (protective) in our GWAS (OR = 0.177, 95% CI [0.083–0.379]), and above 1 (risk) in the UK biobank comparison (1.130, 95% CI [1.004–1.271]), therefore we cannot consider this result validated by the UK biobank data. Other results related to lead variants from our GWAS, namely rs61964606, rs13176661, and rs1331359 were not validated by the UK biobank data, either (*p* > 0.05).

The results of the analysis are contained in the Supplementary Table S5.

### 3.6 Replication Analysis of the Previously Identified Association Signals

Next, we performed a replication analysis of the 12 variants reported to be associated with COVID-19 severity in previous studies. We have chosen associations that emerged in large GWAS meta-studies (Horowitz et al., 2022; Roberts et al., 2022) and recent studies that analyzed associations of *ACE2* genetic variants with COVID-19 severity (Martínez-Gómez et al., 2022; Sabater Molina et al., 2022) (Supplementary Table S6). Our selection approach was focused on variants that showed association with severity and not with susceptibility to COVID-19. Of the 12 selected variants, we replicated 4 variants in phenotype COVID-19 related pneumonia/without pneumonia (the same as severe and moderate versus mild) (*IFNAR2* rs13050728, *LZTFL1* rs35081325, *CCHCR1* rs143334143, *ACE2* rs2106809) and 2 variants in phenotype severe/mild (*LZTFL1* rs35081325, *CCHCR1* rs143334143) at least at nominal *p*-value of 0.05 (Supplementary Table S6). We confirmed that all replicated associations have the same direction of effects (beta) as previously reported.

## 4 Discussion

This study aimed to elucidate genetic risk loci associated with different COVID-19 clinical outcomes in the Serbian population. We detected a novel risk locus at chromosome 13q21.33, significantly associated with the SARS-CoV-2 infection-induced pneumonia. Also, we identified other suggestive signals at positions 3p21.31, 5p15.33, 5q11.2, and 9p23 associated with pneumonia and a suggestive signal at 3p21.31, associated with severe COVID-19 disease.

The peak association signal at 13q21.33 was located close to the gene *KLHL1,* in the intergenic region surrounded by the regulatory sequences. KLHL1 (Kelch Like Family Member 1) is a protein belonging to a family of actin-organizing proteins which modulates voltage-gated calcium channels expressed primarily in various brain tissues (Perissinotti et al., 2014). In a previous genome-wide analysis, the interaction of *KLHL1* locus and early life smoke exposure was found to be associated with the onset of childhood asthma (Sugier et al., 2019). Moreover, it was shown that an increased mutation rate in the *KLHL1* gene was associated with lifetime benzo(a)pyrene exposure in patients with air-pollution-related lung cancers (Yu et al., 2015). Transcriptional factors JUND and FOXA1 binding sites are found in the proximity of the lead variant rs61964606 and both have been related to gene expression regulation in previous studies of SARS-CoV-2 infection (Qiao et al., 2020; Ahmed et al., 2021).

The same locus harbors bidirectional transcripts of the *ATXN8* gene which codes for almost pure polyglutamine protein and the long non-coding antisense *ATXN80S* gene*.* Both genes contain expanded trinucleotide repeats associated with spinocerebellar ataxia type 8 (Batra et al., 2010). Although COVID-19 is primarily a respiratory disease, it can cause neurological manifestations as well. With COVID-19 worldwide expansion, there is an increased number of studies reporting cases of coronavirus induced acute cerebellar ataxia (Chan et al., 2021; Povlow and Auerbach, 2021; Werner et al., 2021). To the best of our knowledge, no previous study connected 13q21.33 nor any of the genes found in this locus to COVID-19. Therefore, we cannot reliably implicate causality without further investigation.

Although 13q21.33 was the only locus to show significant association at the genome-wide level in our study, other loci showed suggestive association. They harbor potentially relevant candidates with a biological function that could be important to the COVID-19 severity.

Our genome-wide analysis of pneumonia and severe COVID-19 confirmed findings from previous studies and showed suggestive signals at chromosome 3p21.31. Locus 3p21.31 has been discovered as the most strongly associated with COVID-19 in a study of severely ill Italian and Spanish patients by the Severe Covid-19 GWAS group (Ellinghaus et al., 2020). This finding was confirmed in another GWAS focused on critically ill patients from the UK intensive care units (Pairo-Castineira et al., 2021). A GWAS by the COVID-19 host genetics initiative (HGI) organized on a worldwide level which included genomic profiles of almost 50,000 SARS-CoV-2 positive, hospitalized, or critically ill COVID-19 patients also showed a strong association of the 3p21.31 locus with severe disease, as well as infection rate (COVID-19 Host Genetics Initiative, 2021). This finding was subsequently confirmed in another large GWAS (Shelton et al., 2021), and also a study that employed a different methodology (Rescenko et al., 2021).

Locus 3p21.31 is rich in protein-coding genes, some of which could impact COVID-19 disease severity. Our genome-wide analysis of pneumonia and severe COVID-19 point to two overlapping regions (defined by linkage disequilibrium analysis), both of which include *SLC6A20* and *LZTFL1* genes. These two genes have been previously considered causative factors related to COVID-19 susceptibility and severity (Ellinghaus et al., 2020; Pairo-Castineira et al., 2021; Shelton et al., 2021). SLC6A20 is an imino acid transporter co-expressed in the intestine and lungs with ACE2 membrane enzyme. Notably, a heterodimer of ACE2 and either SLC6A19 or SLC6A20 serves as a binding site for the SARS-CoV-2 spike protein, which may facilitate viral infection (Camargo et al., 2020). *LZTFL1* gene is implicated in ciliary function in the lungs important for airway viral clearance (Fink-Baldauf et al., 2022). Eight genes (*CCR9, CXCR6, XCR1, CCR3, CCR1, CCR2, CCR5,* and *CCRL2*) related to chemokine pathways, involved in the migration of leukocytes, are located near locus 3p21.31. Elevated levels of chemokines can cause acute respiratory disease syndrome in COVID-19 patients, which is associated with poor outcomes (Khalil et al., 2021).

*EMS1* gene, located at suggestive risk locus 5q11.2, encodes a dermatan sulfate proteoglycan called endocan that is mainly secreted by pulmonary and kidney vascular endothelial cells in response to inflammatory cytokines (Lassalle et al., 1996)**.** Its level can predict multiple organ dysfunctions and mortality in patients with acute respiratory distress (Tang et al., 2014). In one proteomic study, endocan was reported in the top 50 plasma proteins found elevated in the SARS-CoV-2 infected patients with mild to moderate disease (Zhong et al., 2021). Moreover, the *GZMK* gene which codes for granzyme K, a serine protease found in cytoplasmic granules of cytotoxic lymphocytes, is located next to the *EMS1* gene. Decreased level of *GZMK* mRNA, as well as a decreased proportion of effector memory CD8^+^ T cells that produce GZMK, was observed in the peripheral blood of COVID-19 patients compared to healthy subjects (Ramljak et al., 2021). Consistently, patients with severe disease had lower proportions of CD8^+^ T cells that express the *GZMK* gene compared to moderate patients in the study that analyzed COVD-19 single-cell landscape of bronchoalveolar immune cells (Liao et al., 2020).

We identified another suggestive signal at chromosome 5p15.33, ∼300–400 kb distant from genes *IRX4*, *NDUFS6* and *MRPL36.* Exploration of this locus indicated that it may function as an enhancer in different tissues, including the lungs. The *NDUFS6* and *MRPL36* are both nuclear-encoded mitochondrial genes. *NDUFS6* encodes the subunit of the NADH:ubiquinone oxidoreductase (Complex I) while *MRPL36* encodes mitochondrial ribosomal protein. A study that analyzed RNA-Seq data derived from primary cells, cell lines, as well as lung and bronchoalveolar lavage fluid of COVID-19 patients showed that SARS-CoV-2 significantly downregulated nuclear-encoded mitochondrial genes related to cellular respiration and Complex I across all models, while mitochondrial ribosomal protein genes’ expression was particularly downregulated in primary cells (Miller et al., 2021). It has been shown that SARS-CoV-2 proteins directly interact with several Complex I subunits (Gordon et al., 2020). Reports on other respiratory viruses suggested that inhibition of Complex I could promote viral replication (Hu et al., 2019).

The suggestive signal at chromosome 9p23 is located close to genes *TYRP1*, *LURAP1L*, and the antisense transcript *LURAP1L-AS1*. LURAP1L (Leucine Rich Adaptor Protein 1 Like) is a protein predicted to be involved in inflammatory signaling since it is a paralog to LURAP1 which acts as an activator of the canonical NF-κB signaling pathway (Jing et al., 2010). Previous GWA studies identified suggestive associations of *LURAP1L* genetic variants with juvenile idiopathic arthritis (Li et al., 2015) and pulmonary function in smokers (Lutz et al., 2015). *LURAP1L* gene expression was found significantly increased in the CD4^+^ T-cells of obese compared to normal-weighted children with asthma (Rastogi et al., 2018). One study showed that *LURAP1L* was among genes with altered gene expression in lung tissue of deceased COVID-19 patients that could be targeted with the anti-inflammatory activities of glucocorticoid drugs (Sharma, 2021).

The main limitation of this study is its relatively small sample size. This limitation is mitigated by (1) good quality clinical data collected by health professionals, so we did not have to rely on self-reported information from patients, and (2) our study group included only patients hospitalized in the first 3 months of the pandemic, so they were all unvaccinated and likely contracted the same variant of SARS-CoV-2 virus, which limited the number of cofounders. An additional limitation of this study is the absence of the replication cohort. Identified candidate genetic variants need to be validated in an independent group of patients before being recognized as reliable disease markers. If they do get validated, further functional studies can be performed to decipher their biological role in COVID-19 infection.

We have observed that the frequencies of effect alleles of risk loci identified in our study were highly variable among world-wide populations. In order to identify important genetic patterns underlying disease outcomes, it is essential to analyze different ethnicities. Important genetic patterns might be difficult to detect if they are not sufficiently represented in the analyzed population, although they may be much more frequent in other populations. GWAS analysis is not suitable for the identification of rare genetic variants, and this approach has been the most successful so far in the detection of genetic risk factors related to COVID-19.

The current study was the first GWAS to include COVID-19 patients of Serbian origin. In fact, until now, no comprehensive genetic study of COVID-19 patients included patients from southeastern Europe, a region generally underrepresented in genomic research. This study confirms the previously validated genetic locus at 3p21.31 as a marker of severe disease. In addition, our results point to novel genomic loci at 5p15.33, 5q11.2, 9p23, and 13q21.33 potentially implicated in the development of pneumonia and more severe COVID-19 disease.

## Code Availability

We provided two Common Workflow Language (CWL) (Crusoe et al., 2021) workflows that were used on the Seven Bridges Cancer Genome Cloud platform (Lau et al., 2017): (1) a preprocessing pipeline that includes variant calling from the raw microarray data, phasing, and imputation and (2) the GWAS analysis. Both CWL workflows are available as JSON format files in the GitHub repository (https://github.com/markozecevic/covid19gwas).

## Data Availability

GWAS summary data of the current study has been deposited in the National Human Genome Research Institute-European Bioinformatics Institute (NHGRI-EBI) GWAS Catalog database (study accession numbers: severe and moderate/mild - GCST90104347, severe/mild - GCST90104348).

## Supplementary Material

Data Sheet 1 Supplementary Methods.

Data Sheet 2 Supplementary Results.
