# Supplementary material for: Genome-Wide Association Study of COVID-19 Outcomes Reveals Novel Host Genetic Risk Loci in the Serbian Population
Source: Front Genet. 2022 Jul 14;13:911010. doi: 10.3389/fgene.2022.911010 (PMC9329799; doi:10.3389/fgene.2022.911010)
Supplement: Supplementary file 1 [file DataSheet2.pdf]

SUPPLEMENTARY RESULTS

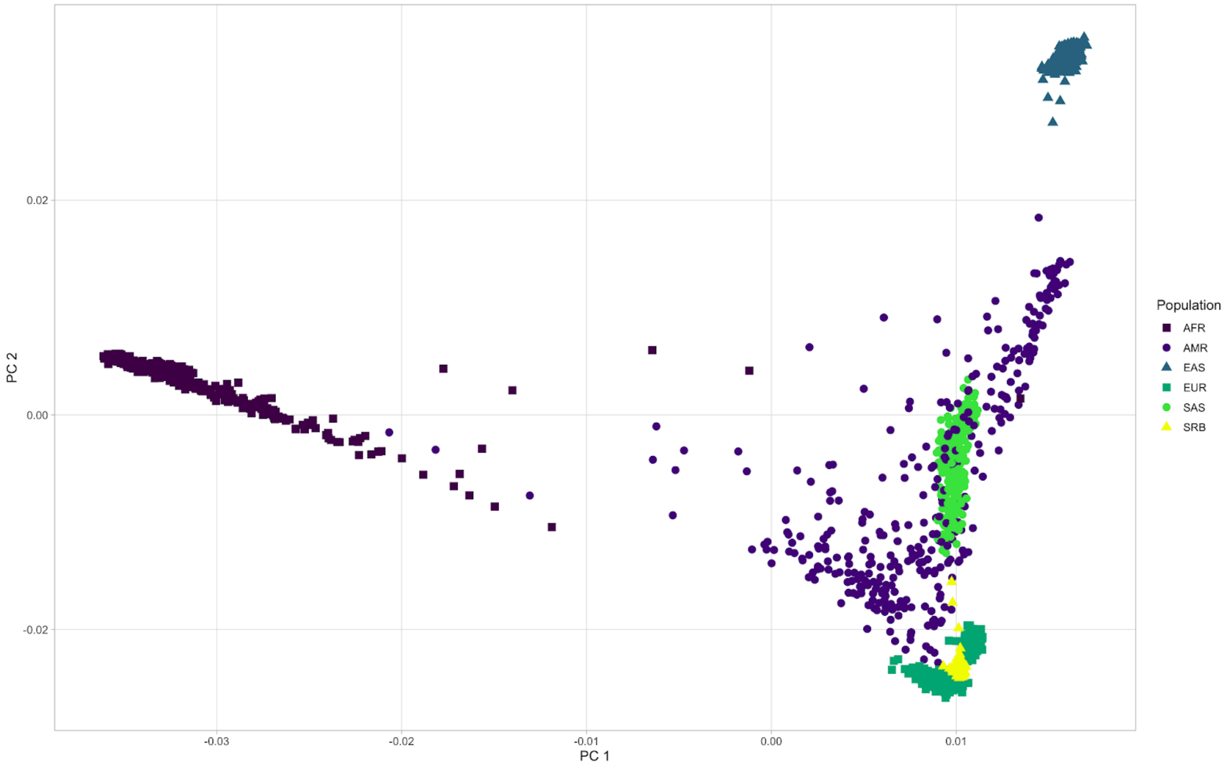

Supplementary Results Figure 1. Principal component analysis of the study group (Serbian COVID-19 patients) together with samples from the 1kGP super-populations. Ancestries: AFR – African, EAS - East Asian, SAS - South Asian, AMR - Ad Mixed American, EUR - European, and SRB - Serbian population.

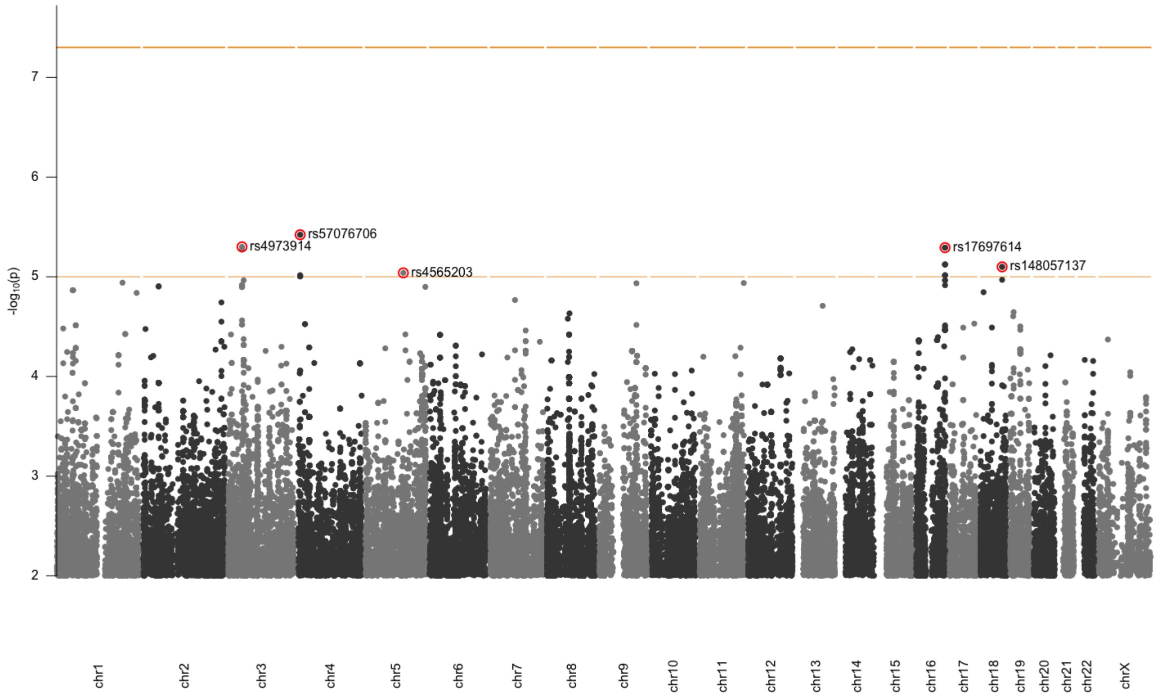

Supplementary Results Figure 2. Genome-wide association of COVID-19 disease severity in Serbian population: severe (n = 34) vs moderate & mild (n = 94).

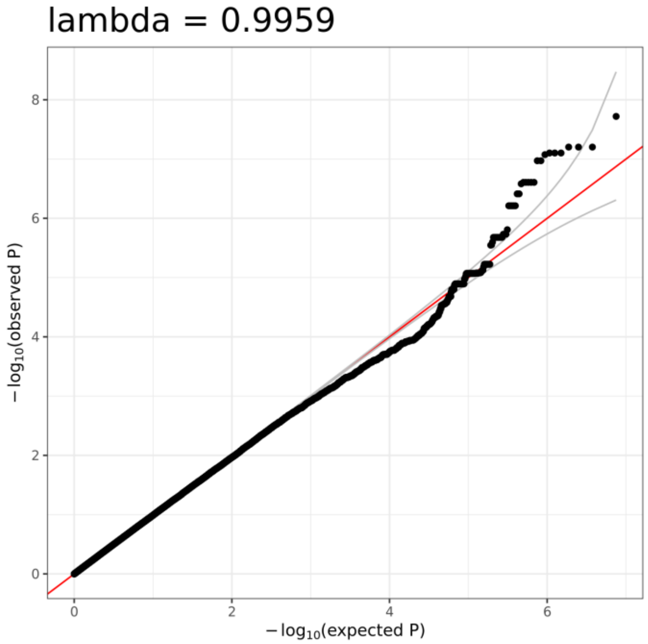

Supplementary Results Figure 3. The relationship of quantile distributions of observed (y-axis) and expected (x-axis) p-values - genome-wide association analysis of COVID-19 disease severity: severe & moderate versus mild.

Supplementary Results Table 1. 95% credible set - plausible causal variants from the 13q21.33 locus – severe & moderate vs mild disease comparison.

PIP	ID	Chr	Pos
0.337	rs61964606	13	70763164
0.054	rs9564670	13	70795676
0.054	rs9529705	13	70795850
0.054	rs9572403	13	70796688
0.045	rs71823498	13	70800379
0.045	rs9564671	13	70804462
0.045	rs9317908	13	70807263
0.042	rs8001442	13	70793406
0.042	rs58597486	13	70794131
0.036	rs12862127	13	70805487
0.022	rs9564668	13	70793950
0.015	rs9529706	13	70801470
0.015	rs7338619	13	70801625
0.015	rs7339068	13	70802009
0.015	rs7339309	13	70802149
0.015	rs9542235	13	70802229
0.014	rs9529700	13	70774297
0.014	rs9529701	13	70774806

0.010	rs2225216	13	70783011
0.010	rs2479942	13	70783660
0.010	rs76300495	13	70785400
0.010	13:70786121:G:T	13	70786121
0.010	rs9317894	13	70763322
0.004	rs2479944	13	70789139
0.004	rs5804497	13	70789589
0.004	rs1410618	13	70789991
0.003	rs9572404	13	70811627
0.003	rs9542229	13	70787303
0.003	rs77174821	13	70786619
0.003	rs7985130	13	70755332
0.003	rs9529687	13	70755853

PIP – posterior inclusion probability; ID – variant identification number; Chr – chromosome; Pos – position

Supplementary Results Table 2. Annotated candidate variants – severe & moderate vs mild disease comparison.

**ID**	**Chr**	**Pos**	**Ref**	**Alt (Effect allele)**	**MAF**	**p-value**	**Odds-ratio**	**Nearest gene**	**Annotation**	**CADD**	**RDB**
rs73060324	3	45785915	G	T	0.08449	7.54E-06	0.084	SACM1L	UTR3	1.974	6
rs73058498	3	45765132	G	T	0.08449	7.54E-06	0.084	SACM1L	intronic	8.65	7
rs140846169	3	45541785	T	C	0.07853	2.99E-02	0.235	LARS2:LARS2-AS1	ncRNA_intronic	0.628	7
rs80163550	3	45551393	G	A	0.07555	2.99E-02	0.235	LARS2	intronic	5.568	5

rs146783234	3	45568854	T	C	0.07555	2.99E-02	0.235	LARS2	intronic	3.26	7
rs73058466	3	45749183	A	G	0.1322	1.48E-04	0.145	SACM1L	intronic	5.014	5
rs73058468	3	45750200	T	C	0.1322	1.48E-04	0.145	SACM1L	intronic	9.73	6
rs140771481	3	45795731	C	CTCTT	0.09046	2.87E-05	0.103	SLC6A20	intergenic	1.255	NA
rs532687890	3	45802715	AC	A	0.1044	6.48E-05	0.121	SLC6A20	intronic	2.505	NA
rs4327428	3	45809329	A	C	0.1103	1.91E-04	0.142	SLC6A20	intronic	1.764	6
rs9818982	3	45812514	A	G	0.1064	1.91E-04	0.142	SLC6A20	intronic	0.185	7
rs17279437	3	45814094	A	G	0.09245	2.87E-05	0.103	SLC6A20	exonic	25.4	5
rs13176661	5	2191105	A	G	0.4294	2.81E-06	0.249	Y_RNA	intergenic	9.098	5
rs11133933	5	2176027	T	C	0.4135	4.17E-04	0.352	Y_RNA	intergenic	5.842	5
rs10053297	5	2178406	C	A	0.4135	4.17E-04	0.352	Y_RNA	intergenic	0.212	5
rs150223040	5	2178612	C	CCCAACA	0.4135	4.17E-04	0.352	Y_RNA	intergenic	1.684	NA
rs10057158	5	2179628	C	T	0.4135	4.17E-04	0.352	Y_RNA	intergenic	1.937	5
rs17584572	5	2179719	C	A	0.4165	4.17E-04	0.352	Y_RNA	intergenic	0.976	5
rs10078052	5	2180108	T	C	0.3996	2.42E-05	0.286	Y_RNA	intergenic	1.868	5
rs10059122	5	2180325	C	T	0.4056	2.42E-05	0.286	Y_RNA	intergenic	0.092	5
rs6555069	5	2182854	T	C	0.4334	3.66E-04	0.351	Y_RNA	intergenic	0.024	5
rs4866649	5	2183448	G	A	0.4533	1.62E-04	0.329	Y_RNA	intergenic	1.911	4
rs13172851	5	2184365	T	C	0.4235	9.54E-06	0.270	Y_RNA	upstream	0.762	5
rs67617651	5	2184774	A	ACT	0.4533	1.62E-04	0.329	Y_RNA	upstream	1.225	NA
rs115633378	5	2184777	A	T	0.4533	1.62E-04	0.329	Y_RNA	upstream	3.474	5

rs11741534	5	2184901	T	C	0.4374	8.35E-06	0.270	Y_RNA	ncRNA_exonic	0.19	5
rs4866652	5	2186956	G	A	0.4165	2.31E-05	0.292	Y_RNA	intergenic	2.706	5
rs6555070	5	2187894	C	G	0.4851	2.73E-04	0.343	Y_RNA	intergenic	0.496	5
rs11743582	5	2188576	C	A	0.4533	1.07E-03	0.391	Y_RNA	intergenic	2.802	7
rs13186652	5	2189102	G	A	0.4145	1.31E-05	0.281	Y_RNA	intergenic	5.199	7
rs6555071	5	2190280	T	A	0.4433	2.12E-04	0.342	Y_RNA	intergenic	2.321	6
rs10462752	5	2190572	A	G	0.4612	5.85E-05	0.305	Y_RNA	intergenic	2.549	5
rs13181974	5	2191339	T	C	0.4294	8.38E-06	0.263	Y_RNA	intergenic	1.827	5
rs116080518	5	2191919	C	A	0.4205	7.88E-06	0.264	Y_RNA	intergenic	0.084	6
rs13188111	5	2192806	T	C	0.4314	8.38E-06	0.263	Y_RNA	intergenic	0.029	5
rs6861764	5	2193662	C	T	0.4533	3.54E-03	0.407	Y_RNA	intergenic	0.135	7
rs4866654	5	2194596	A	G	0.4294	8.38E-06	0.263	Y_RNA	intergenic	0.598	5
rs13157950	5	2197203	C	T	0.4314	8.38E-06	0.263	Y_RNA	intergenic	0.183	5
rs34663818	5	2198824	TTA	T	0.4513	1.38E-04	0.315	Y_RNA	intergenic	0.547	NA
rs6865374	5	2200150	A	G	0.4911	6.59E-05	0.300	Y_RNA	intergenic	0.07	5
rs12189415	5	2200445	C	T	0.4314	8.38E-06	0.263	Y_RNA	intergenic	0.429	5
rs71622161	5	54286050	T	C	0.1819	3.81E-05	0.214	ESM1	intronic	9.518	5
rs78317595	5	54288077	C	T	0.1889	6.59E-06	0.178	ESM1	intronic	10.58	7
rs547882203	5	54288466	T	TA	0.1809	4.16E-05	0.207	ESM1	intronic	0.27	NA
rs10076939	5	54294287	T	C	0.2117	7.07E-05	0.227	ESM1	intronic	0.363	6
rs13183389	5	54294900	T	C	0.1889	6.56E-05	0.216	ESM1	intronic	0.341	6

rs6450276	5	54295109	C	T	0.1948	6.56E-05	0.216	ESM1	intronic	0.634	7
rs6450277	5	54295201	G	A	0.1948	6.56E-05	0.216	ESM1	intronic	2.427	7
rs7036725	9	12340116	T	C	0.08847	1.59E-05	21.930	RNU2-47P	intergenic	1.171	6
rs150842757	9	12340931	ATTCTG	A	0.08847	2.86E-05	19.638	RNU2-47P	intergenic	0.38	NA
rs950850	9	12347681	T	C	0.09443	5.98E-04	9.518	RNU2-47P	intergenic	0.754	6
rs1928877	9	12348879	C	T	0.1292	2.09E-05	9.198	RNU2-47P	intergenic	1.573	NA
rs4741220	9	12351048	T	C	0.1252	2.09E-05	9.198	RNU2-47P	intergenic	1.075	5
rs10756363	9	12351743	G	C	0.1262	2.09E-05	9.198	RNU2-47P	intergenic	6.56	6
9:12354613:A:G	9	12354613	G	A	0.1312	2.09E-05	9.198	RNU2-47P	intergenic	2.527	6
rs2773864	9	12355651	G	A	0.1252	2.09E-05	9.198	RNU2-47P	intergenic	2.198	NA
rs2026329	9	12355858	T	C	0.1252	2.09E-05	9.198	RNU2-47P	intergenic	0.163	6
rs1331350	9	12356017	G	A	0.1272	2.09E-05	9.198	RNU2-47P	intergenic	2.833	7
rs1331351	9	12356314	T	G	0.1511	2.56E-04	4.487	RNU2-47P	intergenic	0.262	7
rs2776997	9	12356597	T	A	0.1153	4.97E-04	6.438	RNU2-47P	intergenic	1.108	7
rs7037091	9	12356679	G	C	0.1252	1.32E-05	10.016	RNU2-47P	intergenic	0.662	7
rs1331359	9	12363456	A	G	0.1302	8.69E-06	0.099	RNU2-47P	intergenic	0.167	6
rs2773847	9	12377233	G	C	0.1362	4.48E-05	0.126	RNU2-47P	intergenic	6.715	5
9:12386201:A:C	9	12386201	A	C	0.1402	4.48E-05	0.126	RNU2-47P	intergenic	0.427	6
9:12386201:C:G	9	12386201	G	C	0.33	4.48E-05	0.126	RNU2-47P	NA	0.401	NA
rs563769168	9	12388603	CT	C	0.1431	2.18E-05	0.128	RNU2-47P	intergenic	1.183	NA
rs2777006	9	12395415	A	T	0.1382	4.48E-05	0.126	RNU2-47P	intergenic	8.527	7

rs2989598	9	12396006	T	A	0.1382	4.48E-05	0.126	RNU2-47P	intergenic	4.165	7
rs2773868	9	12396313	G	T	0.1382	4.48E-05	0.126	RNU2-47P	intergenic	1.852	NA
rs2773867	9	12397700	A	C	0.1392	4.48E-05	0.126	RNU2-47P	intergenic	0.656	NA
rs2777007	9	12397876	G	A	0.1372	4.48E-05	0.126	RNU2-47P	intergenic	4.34	6
rs2777010	9	12398353	C	T	0.1352	4.48E-05	0.126	RNU2-47P	intergenic	1.842	6
rs2773858	9	12402842	G	A	0.1392	2.08E-05	0.119	RNU2-47P	intergenic	3.189	NA
rs147909803	9	12404351	A	ATTCAAGTTTCATATTT	0.1362	1.28E-05	0.111	RNU2-47P	intergenic	6.242	NA
rs1331346	9	12404591	T	C	0.1382	1.28E-05	8.993	RNU2-47P	intergenic	0.24	7
rs2773836	9	12404638	A	G	0.1362	1.28E-05	0.111	RNU2-47P	intergenic	1.482	NA
rs1331347	9	12405051	G	A	0.1362	1.28E-05	0.111	RNU2-47P	intergenic	1.802	6
rs1331348	9	12405316	T	C	0.1382	1.28E-05	0.111	RNU2-47P	intergenic	1.185	5
rs1331349	9	12405391	C	T	0.1382	1.28E-05	0.111	RNU2-47P	intergenic	14.19	5
rs713480	9	12405834	A	T	0.1382	1.28E-05	0.111	RNU2-47P	intergenic	0.961	7
rs1411936	9	12405940	T	C	0.1362	1.28E-05	0.111	RNU2-47P	intergenic	3.458	7
9:12406107:C:G	9	12406107	C	G	0.1362	1.28E-05	0.111	RNU2-47P	intergenic	1.798	7
rs10491748	9	12406559	C	G	0.1362	1.28E-05	0.111	RNU2-47P	intergenic	0.089	7
rs10809770	9	12406786	A	C	0.1362	1.28E-05	0.111	RNU2-47P	intergenic	9.02	6
rs10809771	9	12406989	G	A	0.1382	1.28E-05	0.111	RNU2-47P	intergenic	2.306	7
rs10116714	9	12407578	G	A	0.1382	1.28E-05	0.111	RNU2-47P	intergenic	5.217	6
rs7020457	9	12407774	G	A	0.1362	1.28E-05	0.111	RNU2-47P	intergenic	3.776	7
rs6474707	9	12408246	C	T	0.1362	1.28E-05	0.111	RNU2-47P	intergenic	0.214	6

rs10960662	9	12409469	C	G	0.1362	1.28E-05	0.111	RNU2-47P	intergenic	2.46	7
rs7028923	9	12409518	T	A	0.1382	1.28E-05	0.111	RNU2-47P	intergenic	1.401	7
rs7847412	9	12410088	A	G	0.1382	1.28E-05	0.111	RNU2-47P	intergenic	0.972	7
rs7847547	9	12410173	A	G	0.1382	1.28E-05	0.111	RNU2-47P	intergenic	0.173	7
rs61515516	9	12410624	C	CAAAAA	0.1382	1.28E-05	0.111	RNU2-47P	intergenic	0.904	NA
rs7863850	9	12410979	G	T	0.1362	1.28E-05	0.111	RNU2-47P	intergenic	4.152	6
rs10960667	9	12419134	A	G	0.1362	1.28E-05	0.111	RNU2-47P	intergenic	3.54	6
rs10738275	9	12419639	A	T	0.1362	1.28E-05	0.111	RNU2-47P	intergenic	2.106	7
rs10738276	9	12419825	G	T	0.1362	1.28E-05	0.111	RNU2-47P	intergenic	1.924	6
rs1952986	9	12420496	A	T	0.1332	1.28E-05	0.111	RNU2-47P	intergenic	8.227	7
rs532709834	9	12429645	TAAA	TAAAA	0.4235	1.28E-05	0.111	RNU2-47P	intergenic	NA	NA
rs560822661	9	12429645	TAA	TAAA	0.1392	1.28E-05	0.111	RNU2-47P	intergenic	NA	NA
rs61964606	13	70763164	A	G	0.2008	1.91E-08	10.116	ATXN8OS	intergenic	1.39	7
13:70786121:G:T	13	70786121	G	T	0.159	6.14E-07	9.900	ATXN8OS	intergenic	0.276	6
rs7985130	13	70755332	T	G	0.1262	2.10E-06	0.099	ATXN8OS	intergenic	1.666	5
rs9529687	13	70755853	T	C	0.1262	2.10E-06	0.099	ATXN8OS	intergenic	5.016	7
rs9317889	13	70756729	A	G	0.1262	2.10E-06	0.099	ATXN8OS	intergenic	2.747	7
rs9805140	13	70757063	T	C	0.1262	2.10E-06	0.099	ATXN8OS	intergenic	0.011	7
rs9805141	13	70757108	G	A	0.1262	2.10E-06	0.099	ATXN8OS	intergenic	4.695	6
rs66880880	13	70759085	T	TCA	0.1262	2.10E-06	0.099	ATXN8OS	intergenic	2.535	NA
rs9592683	13	70759905	G	C	0.1322	8.22E-06	0.115	ATXN8OS	intergenic	1.297	7

rs9592684	13	70760123	G	A	0.1262	2.10E-06	0.099	ATXN8OS	intergenic	0.942	6
rs9542203	13	70760265	G	A	0.1262	2.10E-06	0.099	ATXN8OS	intergenic	4.276	7
rs9542204	13	70760365	G	A	0.1262	2.10E-06	0.099	ATXN8OS	intergenic	11.47	6
rs2039085	13	70760697	A	G	0.1262	8.49E-06	0.116	ATXN8OS	intergenic	0.941	NA
rs2039084	13	70760817	T	C	0.1262	8.49E-06	0.116	ATXN8OS	intergenic	5.906	NA
rs9317890	13	70761219	C	T	0.1262	8.49E-06	0.116	ATXN8OS	intergenic	0.999	7
rs9317891	13	70761238	G	C	0.1262	8.49E-06	0.116	ATXN8OS	intergenic	0.049	6
rs9317892	13	70761325	A	T	0.1262	8.49E-06	0.116	ATXN8OS	intergenic	3.753	5
rs9317893	13	70761332	T	C	0.1262	8.49E-06	0.116	ATXN8OS	intergenic	1.752	5
rs2325257	13	70761533	A	G	0.1252	8.49E-06	0.116	ATXN8OS	intergenic	0.559	7
rs9542205	13	70762167	G	T	0.1252	8.49E-06	0.116	ATXN8OS	intergenic	0.432	7
rs9542206	13	70762170	A	G	0.1252	8.49E-06	0.116	ATXN8OS	intergenic	1.21	7
rs9317894	13	70763322	G	A	0.1402	6.14E-07	0.101	ATXN8OS	intergenic	3.345	6
rs9542207	13	70764188	G	A	0.1262	8.49E-06	0.116	ATXN8OS	intergenic	0.424	3a
rs9529688	13	70765346	A	G	0.1262	8.49E-06	0.116	ATXN8OS	intergenic	2.721	5
rs9529689	13	70765557	T	C	0.1262	8.49E-06	0.116	ATXN8OS	intergenic	0.593	6
rs9529690	13	70765842	A	G	0.1252	8.49E-06	0.116	ATXN8OS	intergenic	0.227	7
rs9529692	13	70765999	G	T	0.1252	8.49E-06	0.116	ATXN8OS	intergenic	2.407	6
rs9599571	13	70767444	G	T	0.1252	8.49E-06	0.116	ATXN8OS	intergenic	1.153	6
rs9542214	13	70768684	G	A	0.1252	8.49E-06	0.116	ATXN8OS	intergenic	2.27	6
rs9542219	13	70771341	A	G	0.1233	5.97E-06	0.112	ATXN8OS	intergenic	11.96	7

rs9317903	13	70772656	G	A	0.1233	5.97E-06	0.112	ATXN8OS	intergenic	0.914	7
rs9317904	13	70773055	A	T	0.1233	5.97E-06	0.112	ATXN8OS	intergenic	0.156	7
rs9529700	13	70774297	G	A	0.1382	3.87E-07	0.096	ATXN8OS	intergenic	1.646	7
rs9529701	13	70774806	A	G	0.1382	3.87E-07	0.096	ATXN8OS	intergenic	1.258	6
rs34470346	13	70776519	A	G	0.1213	5.97E-06	0.112	ATXN8OS	intergenic	0.818	7
rs12872111	13	70777044	G	A	0.1143	2.45E-05	0.126	ATXN8OS	intergenic	2.603	6
rs2479941	13	70779853	T	C	0.1243	5.97E-06	8.938	ATXN8OS	intergenic	4.334	7
rs73202510	13	70781671	C	T	0.1272	8.49E-06	0.116	ATXN8OS	intergenic	8.655	7
rs2225216	13	70783011	G	A	0.1412	6.14E-07	9.900	ATXN8OS	intergenic	0.283	7
rs150115021	13	70783490	A	G	0.1272	8.49E-06	0.116	ATXN8OS	intergenic	0.426	7
rs2479942	13	70783660	C	T	0.1412	6.14E-07	9.900	ATXN8OS	intergenic	0.535	7
rs76300495	13	70785400	A	G	0.1412	6.14E-07	9.900	ATXN8OS	intergenic	0.637	6
rs77174821	13	70786619	T	C	0.1412	2.84E-06	8.823	ATXN8OS	intergenic	0.602	6
rs9542229	13	70787303	C	A	0.1412	2.52E-06	8.759	ATXN8OS	intergenic	0.293	7
rs9542231	13	70787448	G	T	0.1252	8.49E-06	0.116	ATXN8OS	intergenic	0.531	6
rs9599579	13	70788871	A	G	0.1233	5.97E-06	0.112	ATXN8OS	intergenic	0.54	6
rs2479944	13	70789139	A	C	0.1382	1.86E-06	9.243	ATXN8OS	intergenic	0.135	6
rs5804497	13	70789589	A	AAAAT	0.1382	1.86E-06	9.243	ATXN8OS	intergenic	0.406	NA
rs1410618	13	70789991	G	A	0.1382	1.86E-06	9.243	ATXN8OS	intergenic	3.723	7
rs9542233	13	70790386	G	A	0.1252	5.97E-06	0.112	ATXN8OS	intergenic	2.711	5
rs9317906	13	70790654	T	G	0.1252	5.97E-06	0.112	ATXN8OS	intergenic	0.055	7

rs9317908	13	70807263	G	T	0.159	6.28E-08	0.102	ATXN8OS	intergenic	3.629	6
rs8001442	13	70793406	G	A	0.1511	1.07E-07	0.091	ATXN8OS	intergenic	5.336	6
rs9564668	13	70793950	T	A	0.165	2.62E-07	0.099	ATXN8OS	intergenic	2.185	7
rs58597486	13	70794131	A	AT	0.1501	1.07E-07	0.091	ATXN8OS	intergenic	0.305	NA
rs9564670	13	70795676	A	G	0.1471	7.91E-08	0.088	ATXN8OS	intergenic	0.732	7
rs9529705	13	70795850	G	A	0.1491	7.91E-08	0.088	ATXN8OS	intergenic	6.804	6
rs9572403	13	70796688	T	C	0.1441	7.91E-08	0.088	ATXN8OS	intergenic	1.641	6
rs71823498	13	70800379	C	CTCTA	0.1571	6.28E-08	0.102	ATXN8OS	intergenic	2.103	NA
rs9529706	13	70801470	T	C	0.1531	2.47E-07	0.112	ATXN8OS	intergenic	15.5	6
rs7338619	13	70801625	A	G	0.1491	2.47E-07	0.112	ATXN8OS	intergenic	1.517	7
rs7339068	13	70802009	T	C	0.1531	2.47E-07	0.112	ATXN8OS	intergenic	1.676	7
rs7339309	13	70802149	G	C	0.1531	2.47E-07	0.112	ATXN8OS	intergenic	1.664	7
rs9542235	13	70802229	A	T	0.1531	2.47E-07	0.112	ATXN8OS	intergenic	4.722	7
rs9564671	13	70804462	A	G	0.1561	6.28E-08	0.102	ATXN8OS	intergenic	0.064	6
rs12862127	13	70805487	C	T	0.159	8.43E-08	0.105	ATXN8OS	intergenic	2.764	7
rs9572404	13	70811627	G	A	0.16	1.56E-06	0.126	ATXN8OS	intergenic	0.417	7
rs9572405	13	70814320	T	C	0.1193	8.32E-06	0.122	ATXN8OS	intergenic	4.004	6
rs60827686	13	70815449	T	C	0.1153	2.76E-05	0.134	ATXN8OS	intergenic	2.454	6
rs61003123	13	70815450	A	G	0.1153	2.76E-05	0.134	ATXN8OS	intergenic	1.733	6
rs9572406	13	70816138	T	G	0.1153	2.76E-05	0.134	ATXN8OS	intergenic	0.786	7
rs550886332	13	70816723	GT	G	0.1193	2.27E-05	0.137	ATXN8OS	intergenic	0.301	NA

rs9564672	13	70816968	T	C	0.1203	8.32E-06	0.122	ATXN8OS	intergenic	1.818	6
rs10650261	13	70819769	AAT	A	0.1153	2.76E-05	0.134	ATXN8OS	intergenic	4.676	NA

ID – variant identification number; Chr – chromosome; Pos – position; Ref – reference allele; Alt – alternative allele; MAF – minor allele frequency; CADD - Combined Annotation Dependent Depletion score of the deleteriousness of single nucleotide variant (the higher the score, the more deleterious the variant is); RDB – Regulome database categorical score (1a-7) of variant functionality based upon its existence in a DNAase hypersensitive site or transcription factor binding site (1a is the highest score with the most biological evidence to be a regulatory element).

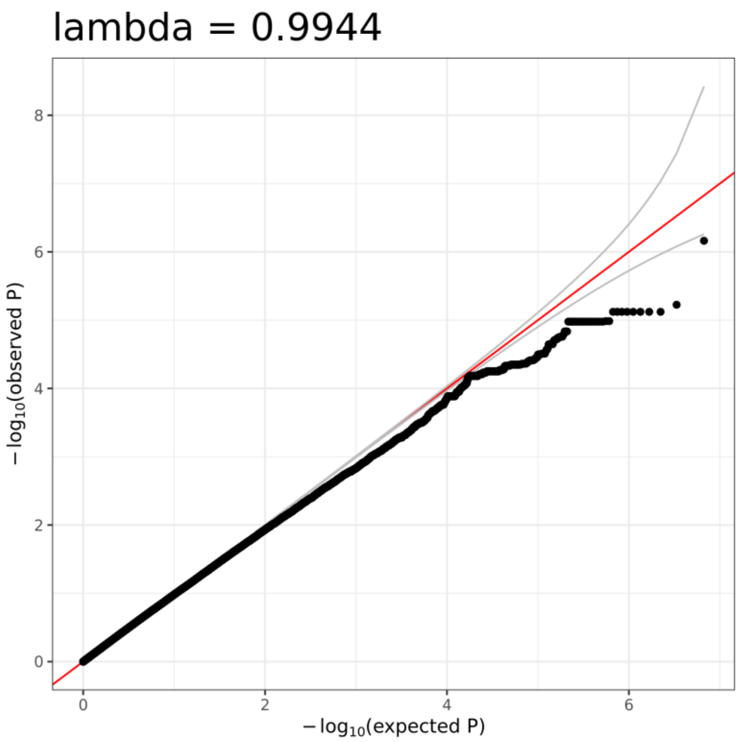

Supplementary Results Figure 4. The relationship of quantile distributions of observed (y-axis) and expected (x-axis) p-values - genome-wide association analysis of COVID-19 disease severity: severe versus mild.

Supplementary Results Table 3. Annotated candidate variants - severe vs mild disease comparison.

ID	Chr	Pos	Ref	Alt (Effect allele)	MAF	p-value	Odds-ratio	Nearest gene	Annotation	CADD	RDB
rs192311430	3	46191413	A	C	0.06262	5.95E-06	45.787	FLT1P1	intergenic	0.27	6

rs73833520	3	46057867	G	A	0.1243	1.30E-04	10.666	XCR1	downstream	1.623	5
rs13089543	3	46183560	G	T	0.1024	4.11E-03	5.677	FLT1P1	downstream	1.837	5
rs13089544	3	46183561	C	T	0.1024	4.11E-03	5.677	FLT1P1	downstream	4.026	5
rs13079478	3	46007823	T	G	0.1223	6.57E-05	11.107	FYCO1	exonic	14.93	5
rs13059238	3	46007825	C	T	0.1233	6.57E-05	11.107	FYCO1	exonic	12.88	5
rs13079869	3	46008087	A	G	0.1223	6.57E-05	11.107	FYCO1	exonic	0.099	5
rs33910087	3	46009487	A	G	0.1223	6.57E-05	11.107	FYCO1	exonic	8.74	5
rs13071283	3	46010007	C	T	0.1243	6.57E-05	11.107	FYCO1	exonic	2.766	2c
rs2230322	3	46063329	C	T	0.1243	1.30E-04	10.666	XCR1	exonic	10.39	NA
rs36023124	3	46039060	C	G	0.1223	6.57E-05	11.107	FYCO1	intergenic	2.021	7
rs71615438	3	46039868	G	A	0.1223	6.57E-05	11.107	FYCO1	intergenic	0.423	6
rs34754340	3	46041837	T	C	0.1223	6.57E-05	11.107	FYCO1	intergenic	6.396	6
rs34836513	3	46046459	A	G	0.1223	6.57E-05	11.107	FYCO1	intergenic	8.658	5
rs34283240	3	46047658	C	G	0.1223	6.57E-05	11.107	FYCO1	intergenic	10.27	5
rs76281521	3	46049764	A	G	0.1223	6.57E-05	11.107	XCR1	intergenic	0.217	5
rs13433997	3	46049765	C	T	0.1272	6.57E-05	11.107	XCR1	intergenic	5.166	5
rs67937868	3	46049827	T	G	0.1252	6.57E-05	11.107	XCR1	intergenic	4.656	5
rs35895243	3	46050568	T	TA	0.1302	6.57E-05	11.107	XCR1	intergenic	6.323	NA
rs55875328	3	46051702	A	G	0.1262	1.30E-04	10.666	XCR1	intergenic	2.562	5
rs67200151	3	46051774	A	G	0.1262	1.30E-04	10.666	XCR1	intergenic	0.182	6
3:46051908:A:G	3	46051908	G	A	0.1262	1.30E-04	10.666	XCR1	intergenic	1.562	7

rs13089855	3	46052263	C	G	0.1262	1.30E-04	10.666	XCR1	intergenic	1.97	4
rs34493660	3	46052800	A	G	0.1262	1.30E-04	10.666	XCR1	intergenic	1.709	7
rs3851346	3	46053581	T	A	0.1243	1.30E-04	10.666	XCR1	intergenic	9.602	7
rs35883205	3	46053968	G	A	0.1243	1.30E-04	10.666	XCR1	intergenic	4.535	3a
rs61650989	3	46054156	T	G	0.1262	1.30E-04	10.666	XCR1	intergenic	2.512	6
rs35669129	3	46055250	A	G	0.1243	1.30E-04	10.666	XCR1	intergenic	1.902	5
rs13081151	3	46055716	A	G	0.1213	7.96E-04	8.179	XCR1	intergenic	0.59	7
rs13081213	3	46055909	T	C	0.1243	1.30E-04	10.666	XCR1	intergenic	1.319	6
rs34168660	3	46056162	A	G	0.1243	1.30E-04	10.666	XCR1	intergenic	1.729	7
rs35751180	3	46056173	T	C	0.1243	1.30E-04	10.666	XCR1	intergenic	0.109	7
rs4362758	3	46056911	A	T	0.1243	1.30E-04	10.666	XCR1	intergenic	0.195	5
rs35921206	3	46057233	A	C	0.1243	1.30E-04	10.666	XCR1	intergenic	1.896	5
rs59166269	3	46070337	G	C	0.1252	1.30E-04	10.666	XCR1	intergenic	2.252	6
rs57437758	3	46070353	C	A	0.1252	1.30E-04	10.666	XCR1	intergenic	4.205	7
rs34562820	3	46071081	A	G	0.1223	1.30E-04	10.666	XCR1	intergenic	8.692	5
rs13082697	3	46071763	C	T	0.1243	1.30E-04	10.666	XCR1	intergenic	10.87	4
rs13060287	3	46071868	G	A	0.1243	1.30E-04	10.666	XCR1	intergenic	9.369	4
rs34619093	3	46072519	A	G	0.1223	1.30E-04	10.666	XCR1	intergenic	6.557	7
rs68087193	3	46072630	T	C	0.1243	1.30E-04	10.666	XCR1	intergenic	4.132	5
rs2102055	3	46072704	A	G	0.1252	1.30E-04	10.666	XCR1	intergenic	7.687	5
rs2102056	3	46072768	A	G	0.1243	1.30E-04	10.666	XCR1	intergenic	0.219	5

rs2088690	3	46072839	T	C	0.1243	1.30E-04	10.666	XCR1	intergenic	4.321	5
rs34774687	3	46073314	C	T	0.1243	1.30E-04	10.666	XCR1	intergenic	3.335	7
rs35814488	3	46073923	G	A	0.1243	1.30E-04	10.666	XCR1	intergenic	3.113	7
rs35467633	3	46074588	GT	G	0.1262	1.30E-04	10.666	XCR1	intergenic	6.59	NA
rs6790866	3	46074711	A	G	0.1243	1.30E-04	10.666	XCR1	intergenic	7.245	6
rs6791016	3	46074858	A	G	0.1243	1.30E-04	10.666	XCR1	intergenic	11.83	7
rs6780028	3	46075090	C	A	0.1243	1.30E-04	10.666	XCR1	intergenic	8.022	6
rs2088692	3	46077332	G	A	0.1243	1.30E-04	10.666	XCR1	intergenic	1.572	5
rs2088693	3	46077390	C	T	0.1441	3.18E-04	7.267	XCR1	intergenic	1.388	5
rs58552652	3	46078292	T	C	0.1441	3.18E-04	7.267	XCR1	intergenic	0.895	7
rs199538350	3	46079524	TTTTTG	T	0.0994	1.30E-04	10.666	XCR1	intergenic	0.525	NA
rs13087776	3	46079871	G	A	0.1441	3.18E-04	7.267	XCR1	intergenic	4.768	7
rs13088143	3	46080024	T	A	0.1441	3.18E-04	7.267	XCR1	intergenic	3.851	6
rs13068410	3	46080176	A	T	0.1441	3.18E-04	7.267	XCR1	intergenic	4.334	5
rs13094262	3	46080912	T	C	0.1441	3.18E-04	7.267	XCR1	intergenic	4.224	NA
rs34805664	3	46081108	T	A	0.1441	3.18E-04	7.267	XCR1	intergenic	5.12	6
rs34438204	3	46081306	C	T	0.1223	1.30E-04	10.666	XCR1	intergenic	5.293	6
rs35028116	3	46081333	C	T	0.1441	3.18E-04	7.267	XCR1	intergenic	5.131	7
rs34610609	3	46081707	T	C	0.1441	3.18E-04	7.267	XCR1	intergenic	2.457	5
rs142722130	3	46082175	CTG	C	0.1441	3.18E-04	7.267	XCR1	intergenic	1.784	NA
rs146279012	3	46082321	AATTGTGCC	A	0.1441	3.18E-04	7.267	XCR1	intergenic	1.756	NA

rs13063033	3	46082481	A	G	0.1223	1.30E-04	10.666	XCR1	intergenic	3.047	6
rs13086534	3	46083201	G	T	0.1441	3.18E-04	7.267	XCR1	intergenic	5.13	7
rs13064632	3	46083357	C	G	0.1441	3.18E-04	7.267	XCR1	intergenic	2.944	5
rs34254987	3	46083634	A	G	0.1441	3.18E-04	7.267	XCR1	intergenic	4.134	6
rs4683162	3	46083941	T	G	0.1451	3.18E-04	7.267	XCR1	intergenic	2.647	7
rs4683163	3	46084372	C	A	0.1441	3.18E-04	7.267	XCR1	intergenic	4.693	7
rs4683164	3	46084461	G	A	0.1441	3.18E-04	7.267	XCR1	intergenic	4.93	6
rs34155121	3	46084742	A	G	0.1441	3.18E-04	7.267	XCR1	intergenic	3.229	7
rs4234453	3	46084949	A	C	0.1441	3.18E-04	7.267	XCR1	intergenic	2.723	5
rs75824352	3	46085208	G	C	0.1441	3.18E-04	7.267	XCR1	intergenic	2.843	7
rs72901034	3	46085321	G	A	0.1441	3.18E-04	7.267	XCR1	intergenic	3.728	6
rs12108042	3	46086083	G	A	0.1441	3.18E-04	7.267	XCR1	intergenic	1.827	6
rs34679077	3	46087992	A	G	0.1223	1.30E-04	10.666	XCR1	intergenic	8.121	3a
rs34867672	3	46088282	C	T	0.1441	3.18E-04	7.267	XCR1	intergenic	0.182	5
rs34924300	3	46088336	T	G	0.1441	3.18E-04	7.267	XCR1	intergenic	1.188	7
rs4683166	3	46089341	C	G	0.1441	3.18E-04	7.267	XCR1	intergenic	3.181	5
rs34386754	3	46090013	A	G	0.1441	3.18E-04	7.267	XCR1	intergenic	0.343	7
rs7652478	3	46090477	A	G	0.1441	3.18E-04	7.267	XCR1	intergenic	5.016	7
rs7642229	3	46090557	G	A	0.1441	3.18E-04	7.267	XCR1	intergenic	3.828	7
rs7642320	3	46090622	G	A	0.1441	3.18E-04	7.267	XCR1	intergenic	6.949	7
rs34863575	3	46094063	G	A	0.1223	1.30E-04	10.666	XCR1	intergenic	10.27	6

rs35772789	3	46095104	G	A	0.1223	1.30E-04	10.666	XCR1	intergenic	7.114	7
rs79298034	3	46095219	G	A	0.1103	1.30E-04	10.666	XCR1	intergenic	0.327	6
rs183817891	3	46095222	T	C	0.09642	1.30E-04	10.666	XCR1	intergenic	2.24	NA
rs558116414	3	46095226	T	C	0.1103	1.30E-04	10.666	XCR1	intergenic	0.275	NA
rs34339943	3	46095336	C	T	0.1223	1.30E-04	10.666	XCR1	intergenic	11.31	5
rs34340587	3	46096043	A	G	0.1223	1.30E-04	10.666	XCR1	intergenic	1.741	6
rs77399277	3	46096798	C	T	0.1223	1.30E-04	10.666	XCR1	intergenic	3.776	6
rs34718164	3	46098526	G	C	0.1223	1.30E-04	10.666	XCR1	intergenic	5.402	6
rs13096741	3	46099154	T	C	0.1223	1.30E-04	10.666	XCR1	intergenic	0.587	5
rs35420565	3	46100138	G	C	0.1223	1.30E-04	10.666	XCR1	intergenic	4.583	7
rs34047915	3	46100491	T	C	0.1223	1.30E-04	10.666	XCR1	intergenic	0.671	4
rs71327014	3	46101466	T	C	0.1223	1.30E-04	10.666	XCR1	intergenic	2.892	5
rs71327015	3	46102173	C	G	0.1223	1.30E-04	10.666	XCR1	intergenic	4.518	7
rs34127208	3	46103680	T	G	0.1223	1.30E-04	10.666	XCR1	intergenic	2.287	3a
rs35516580	3	46107498	G	A	0.1223	1.30E-04	10.666	XCR1	intergenic	1.323	4
rs34766614	3	46107601	G	A	0.1223	1.30E-04	10.666	XCR1	intergenic	7.69	5
rs9824651	3	46108285	T	C	0.1252	1.30E-04	10.666	XCR1	intergenic	3.416	5
rs9825081	3	46108400	A	G	0.1252	1.30E-04	10.666	XCR1	intergenic	0.799	5
rs9877748	3	46111081	G	A	0.1252	1.30E-04	10.666	XCR1	intergenic	6.15	6
rs71327017	3	46111213	C	T	0.1223	1.30E-04	10.666	XCR1	intergenic	1.026	6
rs9845382	3	46111759	A	G	0.1252	1.30E-04	10.666	XCR1	intergenic	9.676	6

rs13069742	3	46114216	G	A	0.1252	1.30E-04	10.666	XCR1	intergenic	10.78	6
rs13072267	3	46116010	A	T	0.1223	1.30E-04	10.666	XCR1	intergenic	7.424	7
rs36057789	3	46117096	G	A	0.1223	1.30E-04	10.666	XCR1	intergenic	3.505	6
rs76647202	3	46117960	G	A	0.1223	1.30E-04	10.666	XCR1	intergenic	2.901	7
rs35951367	3	46118439	C	T	0.1541	2.30E-03	6.279	XCR1	intergenic	4.15	3a
rs71327021	3	46120517	G	A	0.1223	1.30E-04	10.666	XCR1	intergenic	2.561	4
rs13091868	3	46123043	G	A	0.1223	1.30E-04	10.666	XCR1	intergenic	0.618	5
rs13092030	3	46123055	T	C	0.1223	1.30E-04	10.666	XCR1	intergenic	3.004	5
rs34278810	3	46123900	TAC	T	0.1252	1.30E-04	10.666	XCR1	intergenic	1.067	NA
rs17215981	3	46124418	G	A	0.1223	1.30E-04	10.666	XCR1	intergenic	5.459	4
rs13089554	3	46127557	A	G	0.1223	1.30E-04	10.666	FLT1P1	intergenic	5.415	5
rs13095717	3	46128542	A	G	0.1223	1.30E-04	10.666	FLT1P1	intergenic	2.721	5
rs13075528	3	46128709	C	T	0.1223	1.30E-04	10.666	FLT1P1	intergenic	1.834	4
rs13095602	3	46128713	G	A	0.1223	1.30E-04	10.666	FLT1P1	intergenic	0.583	5
rs71327023	3	46131225	G	C	0.1223	1.30E-04	10.666	FLT1P1	intergenic	2.096	3a
rs13063527	3	46135690	A	G	0.1223	1.30E-04	10.666	FLT1P1	intergenic	3.563	5
rs13068570	3	46136438	G	A	0.1223	1.30E-04	10.666	FLT1P1	intergenic	0.058	3a
rs1491950	3	46138107	A	G	0.1223	1.30E-04	10.666	FLT1P1	intergenic	2.19	5
rs4373103	3	46138455	A	G	0.1223	1.30E-04	10.666	FLT1P1	intergenic	0.284	5
rs34492478	3	46138842	T	A	0.1223	1.30E-04	10.666	FLT1P1	intergenic	5.665	7
rs71327024	3	46140073	T	G	0.1223	1.30E-04	10.666	FLT1P1	intergenic	18.33	4

rs1491951	3	46141844	A	G	0.1302	1.30E-04	10.666	FLT1P1	intergenic	1.895	7
rs34460587	3	46142464	T	C	0.1223	1.30E-04	10.666	FLT1P1	intergenic	5.655	5
rs34452002	3	46143187	T	C	0.1223	1.30E-04	10.666	FLT1P1	intergenic	3.236	6
rs34093271	3	46144981	T	G	0.1223	1.30E-04	10.666	FLT1P1	intergenic	11.55	6
rs13093179	3	46146314	T	G	0.1223	1.30E-04	10.666	FLT1P1	intergenic	0.752	4
rs71327025	3	46147504	G	A	0.1223	1.30E-04	10.666	FLT1P1	intergenic	9.595	5
rs35539222	3	46154272	C	T	0.1223	1.30E-04	10.666	FLT1P1	intergenic	4.792	4
rs35110864	3	46154457	A	G	0.1223	1.30E-04	10.666	FLT1P1	intergenic	8.332	7
rs35464221	3	46169040	T	C	0.1243	1.93E-04	8.561	FLT1P1	intergenic	7.735	4
rs35581648	3	46170897	T	G	0.1233	1.93E-04	8.561	FLT1P1	intergenic	0.672	7
rs13085367	3	46172824	C	T	0.1243	1.93E-04	8.561	FLT1P1	intergenic	3.91	6
rs13063635	3	46173072	C	G	0.1243	1.93E-04	8.561	FLT1P1	intergenic	1.634	6
rs71327027	3	46173210	G	T	0.1243	1.93E-04	8.561	FLT1P1	intergenic	4.378	6
rs17282175	3	46173642	T	C	0.1243	1.93E-04	8.561	FLT1P1	intergenic	0.059	4
rs78380307	3	46174331	C	T	0.1213	1.93E-04	8.561	FLT1P1	intergenic	2.328	5
rs17282238	3	46174605	G	A	0.1213	1.93E-04	8.561	FLT1P1	intergenic	3.485	7
rs72092511	3	46176394	A	ATTGTTT	0.1213	1.93E-04	8.561	FLT1P1	intergenic	3.954	NA
rs13068572	3	46177096	A	G	0.1203	1.93E-04	8.561	FLT1P1	intergenic	0.454	5
rs10510748	3	46178538	G	A	0.1024	7.29E-03	5.274	FLT1P1	intergenic	0.405	6
rs7631551	3	46186310	A	C	0.1004	4.11E-03	5.677	FLT1P1	intergenic	1.923	7
rs530312191	3	46194271	TA	T	0.1024	4.11E-03	5.677	FLT1P1	intergenic	0.82	NA

rs71327035	3	46194528	C	T	0.1024	4.11E-03	5.677	FLT1P1	intergenic	0.255	7
rs71327036	3	46194589	A	C	0.1024	4.11E-03	5.677	FLT1P1	intergenic	0.598	7
3:46199680:C:G	3	46199680	C	G	0.1014	4.11E-03	5.677	CCR3	intergenic	1.443	7
rs13086063	3	46200860	C	T	0.1014	4.11E-03	5.677	CCR3	intergenic	2.589	7
rs74532081	3	46200925	A	T	0.1004	4.11E-03	5.677	CCR3	intergenic	1.472	7
rs75549687	3	46200926	C	G	0.1004	4.11E-03	5.677	CCR3	intergenic	0.028	7
rs35482426	3	45914585	C	CTT	0.1223	3.09E-05	13.046	LZTFL1	intronic	3.252	NA
rs34901975	3	45916786	A	G	0.1233	3.09E-05	13.046	LZTFL1	intronic	0.919	5
rs17764831	3	45918215	A	G	0.1233	3.09E-05	13.046	LZTFL1	intronic	1.291	7
rs34518147	3	45920298	C	T	0.1233	1.75E-05	13.835	LZTFL1	intronic	0.397	6
rs74586549	3	45926043	T	C	0.1362	1.75E-05	13.835	LZTFL1	intronic	0.059	4
rs7653372	3	45927759	C	T	0.1402	7.55E-06	16.678	LZTFL1	intronic	4.704	2b
rs34338823	3	45928769	A	G	0.1233	7.55E-06	16.678	LZTFL1:CCR9	intronic	3.755	5
rs57833297	3	45930796	T	TA	0.1282	7.55E-06	16.678	LZTFL1:CCR9	intronic	0.021	NA
rs71325091	3	45932407	A	G	0.1233	7.55E-06	16.678	LZTFL1:CCR9	intronic	6.254	5
rs6782814	3	45936945	C	G	0.1372	7.55E-06	16.678	LZTFL1:CCR9	intronic	0.064	2b
rs17764980	3	45939016	A	G	0.1203	7.55E-06	16.678	LZTFL1:CCR9	intronic	4.193	4
rs17714101	3	45939802	A	G	0.1203	7.55E-06	16.678	LZTFL1:CCR9	intronic	1.892	7
rs17714228	3	45945132	C	A	0.1203	7.55E-06	16.678	LZTFL1	intronic	1.449	7
rs71325092	3	45947018	G	A	0.1203	1.03E-05	15.536	LZTFL1	intronic	2.398	5
rs35280891	3	45951647	A	G	0.1292	6.88E-07	19.845	LZTFL1	intronic	8.293	6

rs34068335	3	45954339	T	C	0.1183	1.03E-05	15.536	LZTFL1	intronic	0.479	5
rs71619614	3	45967800	C	CA	0.1203	6.57E-05	11.107	FYCO1	intronic	1.361	NA
rs2373087	3	45968043	G	T	0.1203	6.57E-05	11.107	FYCO1	intronic	6.279	6
rs35831747	3	45970391	A	G	0.1203	6.57E-05	11.107	FYCO1	intronic	2.765	5
rs13099120	3	45970944	G	C	0.1203	6.57E-05	11.107	FYCO1	intronic	0.456	2b
rs35477280	3	45974092	A	G	0.1203	6.57E-05	11.107	FYCO1	intronic	9.515	7
rs13066516	3	45975443	T	C	0.1203	6.57E-05	11.107	FYCO1	intronic	0.352	6
rs200582580	3	45975485	TA	T	0.1203	6.57E-05	11.107	FYCO1	intronic	1.42	NA
rs77902290	3	45976662	T	C	0.1203	6.57E-05	11.107	FYCO1	intronic	6.755	7
rs147246298	3	45977810	A	AAAG	0.1203	6.57E-05	11.107	FYCO1	intronic	6.23	NA
rs2171531	3	45981171	T	C	0.1213	6.57E-05	11.107	FYCO1	intronic	1.037	4
rs55920693	3	45983476	A	T	0.1223	6.57E-05	11.107	FYCO1:CXCR6	intronic	3.671	5
rs6785091	3	45985347	G	C	0.1252	6.57E-05	11.107	FYCO1:CXCR6	intronic	3.59	6
rs56332428	3	45989873	T	C	0.1223	6.57E-05	11.107	FYCO1	intronic	4.367	4
rs71325095	3	45989921	T	G	0.1223	6.57E-05	11.107	FYCO1	intronic	0.746	4
rs35501575	3	45993645	T	C	0.1213	6.57E-05	11.107	FYCO1	intronic	0.264	5
rs143443576	3	45994205	GGA	G	0.1223	6.57E-05	11.107	FYCO1	intronic	5.338	NA
rs532777636	3	45995708	TGG	T	0.1213	6.57E-05	11.107	FYCO1	intronic	0.973	NA
rs34381952	3	45995748	C	T	0.1223	6.57E-05	11.107	FYCO1	intronic	5.407	6
rs4388012	3	45996501	G	A	0.1223	6.57E-05	11.107	FYCO1	intronic	3.431	5
rs41289616	3	45997263	C	T	0.1223	6.57E-05	11.107	FYCO1	intronic	4.623	7

rs35257780	3	45997506	GA	G	0.1213	6.57E-05	11.107	FYCO1	intronic	11.81	NA
rs147316571	3	45998058	G	GTTC	0.1213	6.57E-05	11.107	FYCO1	intronic	2.172	NA
rs34000569	3	45999209	G	A	0.1213	6.57E-05	11.107	FYCO1	intronic	3.358	4
rs34324101	3	46000728	G	T	0.1213	6.57E-05	11.107	FYCO1	intronic	5.373	5
rs13069079	3	46000870	A	G	0.1213	6.57E-05	11.107	FYCO1	intronic	0.068	5
rs76597151	3	46001227	A	G	0.1223	6.57E-05	11.107	FYCO1	intronic	4.409	5
rs34849862	3	46001367	A	C	0.1213	6.57E-05	11.107	FYCO1	intronic	1.952	7
rs35827997	3	46001720	G	T	0.1223	6.57E-05	11.107	FYCO1	intronic	3.132	5
rs71325096	3	46002963	C	G	0.1213	6.57E-05	11.107	FYCO1	intronic	0.175	6
rs35209528	3	46003496	C	T	0.1213	6.57E-05	11.107	FYCO1	intronic	4.641	4
rs36039366	3	46003537	A	G	0.1223	6.57E-05	11.107	FYCO1	intronic	2.053	4
rs35855315	3	46006239	G	A	0.1233	6.57E-05	11.107	FYCO1	intronic	12	5
rs35525815	3	46006269	T	C	0.1233	6.57E-05	11.107	FYCO1	intronic	3.548	5
rs17280623	3	46006341	G	C	0.1233	6.57E-05	11.107	FYCO1	intronic	10.31	7
rs13078739	3	46007488	A	G	0.1233	6.57E-05	11.107	FYCO1	intronic	7.1	5
rs17214952	3	46011436	G	A	0.1233	6.57E-05	11.107	FYCO1	intronic	4.791	5
rs17215008	3	46012279	C	T	0.1223	6.57E-05	11.107	FYCO1	intronic	4.996	6
rs149942772	3	46012375	C	CTA	0.1233	6.57E-05	11.107	FYCO1	intronic	6.817	NA
rs71325098	3	46012391	G	A	0.1223	6.57E-05	11.107	FYCO1	intronic	4.103	6
rs139901862	3	46012542	TA	T	0.1233	6.57E-05	11.107	FYCO1	intronic	2.146	NA
rs71325100	3	46013832	T	C	0.1223	6.57E-05	11.107	FYCO1	intronic	2.686	5

rs41289622	3	46014545	G	T	0.1223	6.57E-05	11.107	FYCO1	intronic	5.773	4
rs71325101	3	46015569	C	T	0.1233	6.57E-05	11.107	FYCO1	intronic	3.182	7
rs71325102	3	46015933	C	T	0.1233	6.57E-05	11.107	FYCO1	intronic	3.184	5
rs13066062	3	46018344	A	G	0.1223	6.57E-05	11.107	FYCO1	intronic	12.4	3a
rs71615437	3	46018781	G	A	0.08946	9.24E-04	8.278	FYCO1	intronic	5.604	7
rs36122610	3	46022833	A	G	0.1223	6.57E-05	11.107	FYCO1	intronic	7.748	4
rs34442130	3	46024529	A	T	0.1223	6.57E-05	11.107	FYCO1	intronic	6.743	6
rs13075758	3	46025048	A	G	0.1223	6.57E-05	11.107	FYCO1	intronic	4.891	4
rs34044394	3	46029098	C	CA	0.1223	6.57E-05	11.107	FYCO1	intronic	7.654	NA
rs146285477	3	46033167	GCCTTACCCACAGACTCCTTCAC	G	0.1223	6.57E-05	11.107	FYCO1	intronic	2.596	NA
rs17330872	3	46035097	G	A	0.1213	6.57E-05	11.107	FYCO1	intronic	4.383	4
rs142571332	3	46036387	AT	A	0.1282	1.05E-03	7.001	FYCO1	intronic	11.64	NA
rs71327003	3	46036521	T	C	0.1203	6.57E-05	11.107	FYCO1	intronic	7.049	2b
rs60019065	3	46064618	C	G	0.1243	1.30E-04	10.666	XCR1	intronic	2.393	3a
rs71327009	3	46064626	G	C	0.1223	1.30E-04	10.666	XCR1	intronic	7.008	4
rs35930050	3	46065763	A	G	0.1243	1.30E-04	10.666	XCR1	intronic	7.874	2b
rs34584867	3	46065963	A	G	0.1243	1.30E-04	10.666	XCR1	intronic	0.757	5
rs35161099	3	46067507	G	T	0.1223	1.30E-04	10.666	XCR1	intronic	8.762	6
rs35159820	3	46068473	G	A	0.1243	1.30E-04	10.666	XCR1	intronic	9.208	7
rs71327010	3	46068835	T	G	0.1223	1.30E-04	10.666	XCR1	intronic	1.792	5
rs13059906	3	46208858	C	T	0.09841	1.74E-03	7.009	CCR3	intronic	1.354	7

rs34198655	3	46215107	A	G	0.09841	1.74E-03	7.009	CCR3	intronic	0.278	7
rs13089907	3	46215710	T	C	0.09841	1.74E-03	7.009	CCR3	intronic	0.817	7
rs13090848	3	46216084	A	G	0.1004	4.11E-03	5.677	CCR3	intronic	0.875	7
rs149101566	3	46216624	T	C	0.1004	4.11E-03	5.677	CCR3	intronic	2.924	7
rs35342525	3	46216897	C	G	0.1004	4.11E-03	5.677	CCR3	intronic	2.176	7
rs34013035	3	46220682	A	G	0.1004	4.11E-03	5.677	CCR3	intronic	2.783	6
rs143975805	3	46222236	T	C	0.0994	4.11E-03	5.677	CCR3	intronic	1.74	6
rs34059564	3	46226165	C	T	0.1024	5.43E-03	5.321	CCR3	intronic	5.509	2b
rs13061548	3	46226646	C	T	0.1024	5.43E-03	5.321	CCR3	intronic	3.574	6
rs34920132	3	46226769	G	A	0.1024	5.43E-03	5.321	CCR3	intronic	3.493	5
rs68181568	3	46228621	C	CT	0.1044	5.43E-03	5.321	CCR3	intronic	0.362	NA
rs34611049	3	46240826	T	A	0.1034	5.43E-03	5.321	CCR3	intronic	5.958	7
rs3136672	3	46242785	C	T	0.1034	5.43E-03	5.321	CCR3	intronic	0.488	NA
rs57115330	3	46255262	C	T	0.1024	2.49E-03	6.476	CCR3	intronic	0.626	7
rs34498313	3	46255796	C	G	0.1024	2.49E-03	6.476	CCR3	intronic	1.644	7
rs13088766	3	46258226	A	G	0.1024	2.49E-03	6.476	CCR3	intronic	0.113	7
rs11929489	3	46259915	A	C	0.1024	2.49E-03	6.476	CCR3	intronic	2.875	5
rs34985947	3	46260008	T	C	0.1024	2.49E-03	6.476	CCR3	intronic	3.79	5
rs34759782	3	46260444	A	G	0.1024	2.49E-03	6.476	CCR3	intronic	1.93	7
rs13062523	3	46260498	G	C	0.1024	2.49E-03	6.476	CCR3	intronic	0.892	6
rs41537946	3	46260860	T	C	0.1024	2.49E-03	6.476	CCR3	intronic	1.519	7

rs35984988	3	46261256	G	A	0.1024	2.49E-03	6.476	CCR3	intronic	3.071	7
rs34444973	3	46262723	TA	T	0.1024	2.49E-03	6.476	CCR3	intronic	1.354	NA
rs13090194	3	46069573	C	A	0.1243	1.30E-04	10.666	XCR1	upstream	5.714	2b
rs1994491	3	45960420	C	G	0.1193	6.57E-05	11.107	FYCO1	UTR3	4.576	4
rs1994492	3	45960646	C	T	0.1183	6.57E-05	11.107	FYCO1	UTR3	11.76	3a
rs1994493	3	45960700	T	C	0.1183	6.57E-05	11.107	FYCO1	UTR3	9.694	3a
rs75928798	3	45962603	G	A	0.1183	6.57E-05	11.107	FYCO1	UTR3	3.595	4
rs71327006	3	46058908	A	G	0.1223	1.30E-04	10.666	XCR1	UTR3	1.746	7
rs71327007	3	46059249	T	C	0.1223	1.30E-04	10.666	XCR1	UTR3	7.083	4
rs35454877	3	46059484	C	T	0.1243	1.30E-04	10.666	XCR1	UTR3	1.554	4
rs35334665	3	46061218	C	T	0.1223	1.30E-04	10.666	XCR1	UTR3	0.881	5
rs36040135	3	46061304	G	A	0.1243	1.30E-04	10.666	XCR1	UTR3	5.338	4
rs13074382	3	46061840	G	A	0.1243	1.30E-04	10.666	XCR1	UTR3	4.761	5
rs13097556	3	46061997	C	T	0.1243	1.30E-04	10.666	XCR1	UTR3	1.161	5
rs876668	3	46063753	C	T	0.1243	1.30E-04	10.666	XCR1	UTR5	1.135	NA

ID – variant identification number; Chr – chromosome; Pos – position; Ref – reference allele; Alt – alternative allele; MAF – minor allele frequency; CADD - Combined Annotation Dependent Depletion score of the deleteriousness of single nucleotide variant (the higher the score, the more deleterious the variant is); RDB – Regulome database categorical score (1a-7) of variant functionality based upon its existence in a DNAase hypersensitive site or transcription factor binding site (1a is the highest score with the most biological evidence to be a regulatory element).

Supplementary Results Table 4. Distribution of risk effect alleles across Serbian and 1kGP populations

Variant ID	COVID-19 phenotype	chr	Effect Allele	AFR	EAS	SAS	AMR	CEU	FIN	GBR	IBS	TSI	SRB	dAF
**rs61964606**	pneumonia	13	G	0.439	0.617	0.794	0.752	0.813	0.778	0.797	0.804	0.804	0.824	**0.385**
rs73058498	pneumonia	3	T	0.998	1.000	0.981	0.955	0.914	0.904	0.890	0.925	0.939	0.918	0.110
**rs13176661**	pneumonia	5	G	0.914	0.681	0.790	0.537	0.611	0.581	0.511	0.584	0.561	0.496	**0.418**
rs78317595	pneumonia	5	T	0.986	0.991	0.922	0.893	0.803	0.899	0.775	0.799	0.780	0.777	0.216
**rs1331359**	pneumonia	9	G	0.949	0.465	0.838	0.576	0.859	0.879	0.802	0.930	0.869	0.880	**0.484**
**rs35280891**	severe disease	3	A	0.006	0.004	0.369	0.063	0.121	0.207	0.121	0.075	0.126	0.116	**0.365**

dAF - genetic variability among populations for each risk loci was calculated by subtracting the maximum and the minimum effect allele frequency across analyzed population groups. Bolded are loci with high variance across world-wide populations.

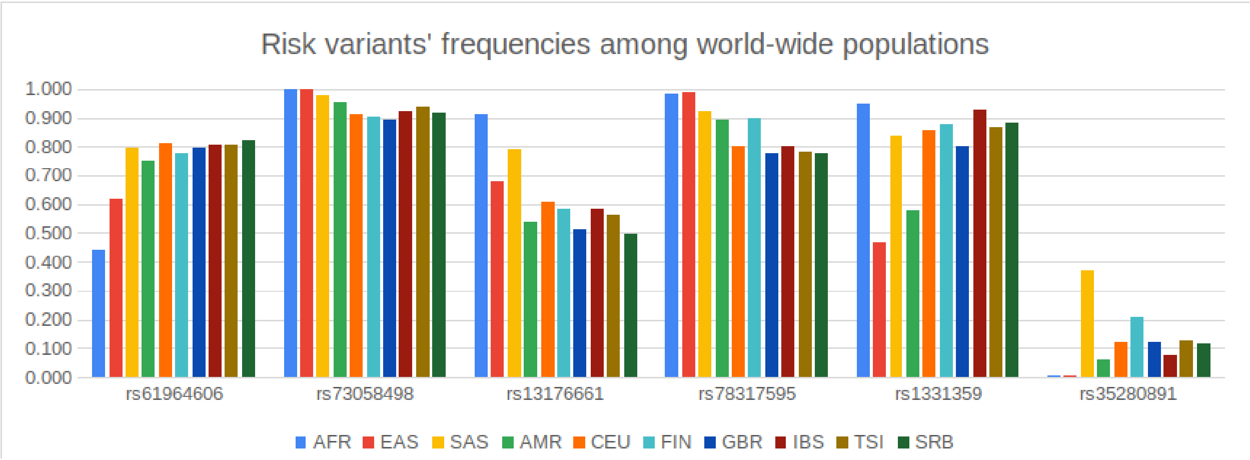

Supplementary Results Figure 5. Frequencies of the lead variants in the world-wide populations extracted from the 1000 Genome Project. Populations: AFR – African, EAS - East Asian, SAS - South Asian, AMR – Ad Mixed American, CEU - Utah residents with Northern and Western European ancestry, FIN - Finnish in Finland, GBR - British in England and Scotland, IBS - Iberian populations in Spain, TSI - Tuscany in Italy, and SRB - Serbian population.

Supplementary Results Table 5. Validation analysis of variants associated with disease severity in the current GWAS using UK biobank data from European population

Variant			Current GWAS statistics				UKBB statistics: severe vs. non-severe COVID positive. cases/controls: 1,120 / 14,695				UKBB statistics: hospitalized positive vs. non-hospitalized positive. cases/controls: 2,884 / 13,667			
Lead variant	Nearest gene(s)	Effect allele	p value	OR	95% CI low	95% CI high	p value	OR	95% CI low	95% CI high	p value	OR	95% CI low	95% CI high
rs61964606	*KLHL1, ATXN8, ATXN80S*	G	1.91×10^{-8}	10.115	4.458	22.949	0.951	0.997	0.891	1.114	0.212	0.954	0.886	1.027
rs73060324	*SACM1L*	G	7.54×10^{-6}	0.084	0.028	0.257	0.477	0.946	0.811	1.103	0.0745	0.912	0.824	1.009
rs13176661	*IRX4, NDUFS6, MRPL36*	A	2.81×10^{-6}	0.249	0.138	0.449	0.522	0.971	0.886	1.064	0.762	0.991	0.933	1.053
rs78317595	*ESM1*	C	6.59×10^{-6}	0.177	0.083	0.379	**0.042**	1.130	1.004	1.271	0.221	1.050	0.971	1.136
rs1331359	*TYRP1, LURAP1L*	A	8.69×10^{-6}	0.099	0.035	0.279	0.324	1.070	0.935	1.225	0.537	1.028	0.940	1.125
rs35280891	*LZTFL1*	A	6.88×10^{-7}	19.846	5.728	68.761	**2.22×10^{-4}**	1.328	1.143	1.545	**7.57×10^{-7}**	1.287	1.164	1.422

OR – odds ratio; CI low and CI high – lower and upper bound of the odds ratio confidence interval. UKBB – UK biobank

Supplementary Results Table 6. Replication analysis of previously reported genetic variants associated with COVID-19 severity on the current study data

Discovery study	Genetic variant	Discovery study reported beta	Discovery study effect allele	Current study phenotype	Current study effect allele	p value	Beta	OR	95% CI low	95% CI high
HGI B2 EUR Severity	*IGF1* rs10860891	0.23	C	Pneumonia vs Without Pneumonia	A	0.652	-0.205	0.815	0.333	1.991
HGI B2 EUR Severity	*OAS3* rs7310667	-0.15	A	Pneumonia vs Without Pneumonia	G	0.976	-0.011	0.989	0.473	2.067
Horowitz et al. Hospitaled vs. Negative/Unknown	*KAT7* rs9903642	0.27	A	Pneumonia vs Without Pneumonia	A	0.366	0.88	2.411	0.357	16.297
HGI B2 EUR Severity	*DPP9* rs2277732	0.21	A	Pneumonia vs Without Pneumonia	A	0.501	-0.223	0.800	0.418	1.531
**HGI B2 EUR Severity**	***IFNAR2*** **rs13050728**	**0.18**	**T**	**Pneumonia vs Without Pneumonia**	**A**	**0.035**	**-0.64**	**0.527**	**0.291**	**0.957**
Horowitz et al. Hospitaled vs. Negative/Unknown	*TMPRSS2* rs2298661	-0.14	A	Pneumonia vs Without Pneumonia	A	0.279	-0.379	0.685	0.345	1.359
**HGI B2 EUR Severity**	***LZTFL1*** **rs35081325**	**0.6**	**T**	**Pneumonia vs Without Pneumonia**	**T**	**0.00029**	**1.905**	**6.719**	**2.406**	**18.766**
**Horowitz et al. Severe vs. Negative or**	***CCHCR1***	**0.28**	**A**	**Pneumonia vs Without**	**A**	**0.037**	**0.965**	**2.625**	**1.055**	**6.530**

**Unknown**	**rs143334143**			**Pneumonia**						
HGI B2 EUR Severity	*STM2A* rs622568	0.23	C	Pneumonia vs Without Pneumonia	C	0.673	-0.174	0.840	0.373	1.892
Horowitz et al. Positive vs. Negative	*SLC6A20* rs2531743	-0.06	G	Pneumonia vs Without Pneumonia	A	0.473	-0.231	0.794	0.423	1.489
**Molina et al. Severity**	***ACE2*** **rs2106809**	**2.433**	**recessive model (GG)**	**Pneumonia vs Without Pneumonia**	**G**	**0.030**	**0.699**	**2.012**	**1.064**	**3.804**
Molina et al. Severity	*ACE2* rs2285666	2.53	recessive model (TT)	Pneumonia vs Without Pneumonia	T	0.067	0.582	1.790	0.960	3.338
HGI B2 EUR Severity	*IGF1* rs10860891	0.23	C	Severe vs Mild	A	0.780	0.188	1.207	0.321	4.540
HGI B2 EUR Severity	*OAS3* rs7310667	-0.15	A	Severe vs Mild	G	0.484	-0.383	0.682	0.233	1.992
Horowitz et al. Hospitaled vs. Negative/Unknown	*KAT7* rs9903642	0.27	A	Severe vs Mild	A	0.838	-0.226	0.798	0.091	7.012
HGI B2 EUR Severity	*DPP9* rs2277732	0.21	A	Severe vs Mild	A	0.136	-0.726	0.484	0.186	1.257
HGI B2 EUR Severity	*IFNAR2* rs13050728	0.18	T	Severe vs Mild	A	0.520	-0.301	0.740	0.296	1.852
Horowitz et al. Hospitaled vs.	*TMPRSS2*	-0.14	A	Severe vs Mild	A	0.416	-0.398	0.672	0.258	1.751

Negative/Unknown	rs2298661									
**HGI B2 EUR Severity**	***LZTFL1*** **rs35081325**	**0.6**	**T**	**Severe vs Mild**	**T**	**$1.05x10^{-5}$**	**3.372**	**29.137**	**6.739**	**125.980**
**Horowitz et al. Severe vs. Negative or Unknown**	***CCHCR1*** **rs143334143**	**0.28**	**A**	**Severe vs Mild**	**A**	**0.018**	**1.517**	**4.559**	**1.293**	**16.075**
HGI B2 EUR Severity	*STM2A* rs622568	0.23	C	Severe vs Mild	C	0.267	-0.615	0.541	0.183	1.601
Horowitz et al. Positive vs. Negative	*SLC6A20* rs2531743	-0.06	G	Severe vs Mild	A	0.818	0.108	1.114	0.443	2.804
Molina et al. Severity	*ACE2* rs2106809	2.433	recessive model (GG)	Severe vs Mild	G	0.070	0.835	2.305	0.932	5.700
Molina et al. Severity	*ACE2* rs2285666	2.53	recessive model (TT)	Severe vs Mild	T	0.151	0.644	1.904	0.790	4.591

OR – odds ratio; CI low and CI high – lower and upper bound of the odds ratio confidence interval
