# Supplementary material for: Genome-Wide Association Study of COVID-19 Outcomes Reveals Novel Host Genetic Risk Loci in the Serbian Population
Source: Front Genet. 2022 Jul 14;13:911010. doi: 10.3389/fgene.2022.911010 (PMC9329799; doi:10.3389/fgene.2022.911010)
Supplement: Supplementary file 2 [file DataSheet1.PDF]

# SUPPLEMENTARY METHODS

This material has the objective to give readers additional information about data processing, quality control and genome wide association analysis. The titles of the paragraphs correspond to specific Material and Methods section parts that they explain.

## 1. Data preprocessing, variant calling and imputation

### 1.1 Cluster file generation

For inferring cluster positions that are representative of the overlying population, at least 100 samples are necessary (Illumina (2016). Infinium Genotyping Data Analysis. [Technical Data Sheet]. Retrieved on September 1st, 2021 from http://www.illumina.com/Documents/products/technotes/technote_infinium_genotyping_data_analysis.pdf) which is in agreement with our total genotyping group size (216 samples). Initial genotype calling from GSAMD intensity data (IDAT) files and quality control (QC) analysis before cluster file generation was performed using GenomeStudio v.2.0 software with GSAMD-24v3-0-EA_20034606_A1.bpm manifest file based on the human genome assembly hg19.

To create a cluster file, first a series of QC procedures were applied in sequential order (Guo et al., 2014; Zhao et al., 2018): (1) raw IDAT files were loaded into GenomeStudio and automatic clustering was performed, (2) samples with call rate < 0.95 were filtered out and data was re-clustered as follows: autosomal, pseudoautosomal and mitochondrial DNA (mtDNA) loci were re-clustered using all samples, X-linked loci using female samples and Y-linked loci using male samples only; (4) X-linked, mtDNA and Y loci clusters were visually inspected and manually edited or zeroed if clusters were not in agreement with haploid genome expectations; (5) all loci were evaluated for cluster quality metrics (loci with GenTrain score < 0.7 and/or cluster separation ≤ 0.3 and/or intensity of heterozygous clusters AB T Mean < 0.2 and > 0.8 were visually inspected and curated, while loci with AB R Mean ≤ 0.2 were zeroed), (6) loci with heterozygosity falling outside of the -0.4 to 0.4 range were excluded, (7) variants with minor frequency < 0.1 were sorted and the first several hundred manually reviewed, and finally, (8) variants with call frequency < 0.95 were reviewed and manually re-clustered if possible or filtered out from further analysis. At the end of the QC procedure, a cluster file for the Serbian population was generated and exported from GenomeStudio, as well as lists of samples and variants that passed QC. Out of 216 samples, 16 did not achieve the required quality threshold (> 0.95 call rate) and were excluded from further analysis. For this study, we selected autosomal, X, and mtDNA loci, which at this point included 722,178 variants after the initial QC analysis.

As an additional QC measure, we compared the allele frequencies from our dataset against the publicly available Phase-3 1000 Genome Project (1kGP) reference panel for the European super-population. The absolute differences of allele frequencies were calculated and sorted in order to identify large differences in two datasets (> 0.2) which could indicate incorrectly clustered variants that should be reviewed or filtered out. Extreme outliers compared to 1kGP data were visually

inspected in GenomeStudio and 4 variants were excluded from further analysis after we found that the clusters for these SNPs were not clean.

## 1.2 Variant calling and imputation

To perform GWAS on a wider set of variants, we have developed a preprocessing pipeline that encompasses variant calling, phasing, and imputation. Illumina's GenCall algorithm, available through the IAAP Genotyping Command Line Interface v1.1 (iaap-cli) was used to call variants from raw IDAT (Intensity DATa) files with a custom EGT cluster file that we had created previously. The Resulting GTC files were converted to VCF using the BCFtools gtc2vcf plugin (github.com/freeseek/gtc2vcf). The variants with a call rate below 0.95 across the 128 patients cohort and a Hardy–Weinberg equilibrium (HWE) p-value $< 1 \times 10^{-10}$ were filtered out using VCFtools v0.1.14 (Danecek et al., 2011). VCFtools was also used to count the number of homozygous sites and calculate the inbreeding coefficient FHET for every sample in our dataset. None of the samples had a high inbreeding coefficient (max absolute FHET = 0.04748) so no filtering was performed at this step. Haplotype estimation was performed using SHAPEIT4 v4.2.1 (Delaneau et al., 2019) using a hg19-based set of human genetic maps that is distributed together with the software. Minimac4 v1.0.2 (Fuchsberger et al., 2015) was used for estimation of missing genotypes and a 1kGP Phase 3 (version 5) set of human reference panels was used as a reference. Variants with a low imputation quality score ($r^2 < 0.3$) were filtered out using BCFtools 1.9 (Danecek et al., 2021). Additional auxiliary tools were also included in the pipeline for actions such as variant call file format and content checks (EBI vcf-validator v0.7), indexing and manipulation (BCFtools, VCFtools, Tabix v1.9.0).

## 2. Genome-wide association study

The GWAS analysis pipeline was based on the GENESIS v2.10.0 (Gogarten et al., 2019) R/Bioconductor package and the various methods it implements. It has provided us with a mixed model framework that accounts for genetic relatedness and allows for the inclusion of different risk factors as covariates.

We analyzed the ancestry of our study group using the principal component analysis of a merged dataset containing our 128 samples and 2504 samples from 1kGP. 1kGP data was pre-processed with BCFtools 1.15.1, LD-pruned and merged with our data using Plink 1.9 (Chang et al., 2015). A total of 425,451 common variants between our dataset and the LD-pruned 1kGP dataset were used to calculate the principal components, also using Plink.

In order to evaluate relatedness and population structure while avoiding having results driven by clusters of variants in high LD regions of the genome - LD pruning was performed. All pairs of markers within a moving window of 10 Mb were compared with each other to measure their pairwise LD and a panel of 174,962 common (MAF > 0.01) and independent (pairwise $r^2 < 0.1$) variants were chosen to perform the population structure and relatedness analysis.

We analyzed the ancestry of our study group using the principal component analysis of a merged dataset containing our 128 sample dataset and 2504 1kGP samples. 1kGP data was pre-processed with BCFtools 1.15.1, LD-pruned and merged with our data using Plink 1.9. A total of 425,451

common variants between our dataset and the LD-pruned 1kGP dataset were used to calculate the principal components, also using Plink.

The KING robust algorithm, available in the SNPRelate v1.24.0 R/Bioconductor package was then used on pruned variants only, in order to estimate the initial kinship coefficients for each pair of samples (Zheng et al., 2012). These kinship estimates represent a measure of ancestry divergence that the PC-AiR algorithm accounted for while computing ancestry principal components (PCs). Related individuals in our dataset have been identified by having a kinship coefficient higher than the default threshold of 0.0442 representing third-degree relatives or closer. The population structure was determined on an unrelated subset of the LD-pruned dataset. Finally, using PC-Relate, we re-estimated kinship coefficients to get an estimate of the family structure while accounting for population structure by conditioning on the top 3 ancestry PCs generated by PC-AiR.

After we have obtained the kinship coefficient matrix from PC-Relate, we fitted a generalized linear mixed model (GLMM) containing both fixed effects (independent covariates) and random effect which models the genetic correlation between the individuals (kinship matrix), under the null hypothesis of no genotype effects. We did not include any of the computed ancestry PCs as fixed effects in the model as we did not expect a substantial population substructure and the pairwise scatter plots of the first 6 PCs showed a lack of clustering. Previous studies have identified several independent risk factors, including age and male sex (Hu and Wang, 2021). In our model formula, we have included age and sex as well as an interaction term (age*sex) between the two. A GLMM of the following form was considered (Chen et al., 2016):

$$\mathrm{logit}(\mu_i) = \mathbf{X}_i\boldsymbol{\alpha} + G_i\beta + b_i$$

where $\mu_i$ is the probability of the binary phenotype (i.e. severe COVID-19 or having pneumonia) given the covariates $\mathbf{X}_i$ (age, sex and age*sex), genotype $G_i$ and the random effect $b_i$. The vector $\boldsymbol{\alpha}$ represents fixed covariate effects and $\beta$ is the genotype effect. It was assumed that $\mathbf{b} \sim N(0,\tau\mathbf{V})$ where $\mathbf{V}$ is the genetic relatedness matrix and $\tau$ is the variance component. The null model, which is the same for all variants and is fitted only once, is of the form:

$$\mathrm{logit}(\mu_{i0}) = \mathbf{X}_i\boldsymbol{\alpha} + b_i$$

The fitted null model was then used for single variant association testing and score tests were performed for all variants with MAC >= 10 individually. We have chosen to apply the saddle point approximation (SPA) to the score test statistic to estimate the null distribution. SPA is recommended for binary outcomes, particularly for rarer variants in datasets with case-control imbalance (Zhou et al., 2018).

Again, several auxiliary tools and custom scripts for variant call file manipulation (BCFtools, SnpSift v4.3 (Cingolani et al., 2012)), format conversion, and visualization (LocusZoom Standalone v1.4) have been included in the pipeline. Manhattan plots have been created using the karyoploteR v1.18.0 R/Bioconductor package (Gel and Serra, 2017). The two thresholds highlighted are those of genome-wide significance ($p < 5 \times 10^{-8}$) - representing a Bonferroni-

corrected 5% family-wise error rate threshold for the estimated effective number of 1,000,000 independent common genetic variants given the linkage disequilibrium structure of the human genome (Uffelmann et al., 2021); and suggestive association ($p < 1 \times 10^{-5}$) - a less stringent threshold intended for the identification of SNPs that should be considered in follow-up studies.

# 3 Post-GWAS analysis

## 3.1 Variant annotation in FUMA software

LD clumping and variant annotation were performed using the FUMA v1.3.7 web application (Watanabe et al., 2017). First, a list of 'independent significant' variants passing the suggestive association threshold ($p < 1 \times 10^{-5}$) and independent from each other at the linkage disequilibrium (LD) threshold $r^2 < 0.6$ was generated. These variants were chosen following a greedy algorithm that at each step chooses the variant with the smallest p-value and removes variants that are in LD with the chosen variant from the selection set. 'Lead variants' are a subset of independent significant variants that are independent of each other at LD threshold $r^2 < 0.1$. Independent significant variants that are physically close (< 250 kb) define a 'genomic risk locus'. Variant annotation for each locus was made on independent significant variants and all the variants in LD with them that are less than 250 kb away, and with a score test p-value < 0.05.

# REFERENCES


Chang, C. C., Chow, C. C., Tellier, L. C., Vattikuti, S., Purcell, S. M., and Lee, J. J. (2015). Second-generation PLINK: rising to the challenge of larger and richer datasets. *Gigascience* 4, 7. doi:10.1186/s13742-015-0047-8.

Chen, H., Wang, C., Conomos, M. P., Stilp, A. M., Li, Z., Sofer, T., et al. (2016). Control for Population Structure and Relatedness for Binary Traits in Genetic Association Studies via Logistic Mixed Models. *Am. J. Hum. Genet.* 98, 653–66. doi:10.1016/j.ajhg.2016.02.012.

Cingolani, P., Platts, A., Wang, L. L., Coon, M., Nguyen, T., Wang, L., et al. (2012). A program for annotating and predicting the effects of single nucleotide polymorphisms, SnpEff: SNPs in the genome of Drosophila melanogaster strain w1118; iso-2; iso-3. *Fly (Austin).* 6, 80–92. doi:10.4161/fly.19695.

Danecek, P., Auton, A., Abecasis, G., Albers, C. A., Banks, E., DePristo, M. A., et al. (2011). The variant call format and VCFtools. *Bioinformatics* 27, 2156–8. doi:10.1093/bioinformatics/btr330.

Danecek, P., Bonfield, J. K., Liddle, J., Marshall, J., Ohan, V., Pollard, M. O., et al. (2021). Twelve years of SAMtools and BCFtools. *Gigascience* 10, giab008. doi:10.1093/gigascience/giab008.

Delaneau, O., Zagury, J. F., Robinson, M. R., Marchini, J. L., and Dermitzakis, E. T. (2019). Accurate, scalable and integrative haplotype estimation. *Nat. Commun.* 10. doi:10.1038/s41467-019-13225-y.

Fuchsberger, C., Abecasis, G. R., and Hinds, D. A. (2015). Minimac2: Faster genotype imputation. *Bioinformatics* 31, 782–784. doi:10.1093/bioinformatics/btu704.

Gel, B., and Serra, E. (2017). KaryoploteR: An R/Bioconductor package to plot customizable genomes displaying arbitrary data. *Bioinformatics* 33, 3088–3090. doi:10.1093/bioinformatics/btx346.

Gogarten, S. M., Sofer, T., Chen, H., Yu, C., Brody, J. A., Thornton, T. A., et al. (2019). Genetic association testing using the GENESIS R/Bioconductor package. *Bioinformatics* 35, 5346–5348. doi:10.1093/bioinformatics/btz567.

Guo, Y., He, J., Zhao, S., Wu, H., Zhong, X., Sheng, Q., et al. (2014). Illumina human exome genotyping array clustering and quality control. *Nat. Protoc.* 9, 2643–2662. doi:10.1038/nprot.2014.174.

Hu, J., and Wang, Y. (2021). The Clinical Characteristics and Risk Factors of Severe COVID-19. *Gerontology* 67, 255–266. doi:10.1159/000513400.

Uffelmann, E., Huang, Q. Q., Munung, N. S., de Vries, J., Okada, Y., Martin, A. R., et al. (2021). Genome-wide association studies. *Nat. Rev. Methods Prim.* 1, 59. doi:10.1038/s43586-021-00056-9.

Zhao, S., Jing, W., Samuels, D. C., Sheng, Q., Shyr, Y., and Guo, Y. (2018). Strategies for processing and quality control of Illumina genotyping arrays. *Brief. Bioinform.* 19, 765–775. doi:10.1093/bib/bbx012.

Zheng, X., Levine, D., Shen, J., Gogarten, S. M., Laurie, C., and Weir, B. S. (2012). A high-performance computing toolset for relatedness and principal component analysis of SNP data. *Bioinformatics* 28, 3326–3328. doi:10.1093/bioinformatics/bts606.

Zhou, W., Nielsen, J. B., Fritsche, L. G., Dey, R., Gabrielsen, M. E., Wolford, B. N., et al. (2018). Efficiently controlling for case-control imbalance and sample relatedness in large-scale genetic association studies. *Nat. Genet.* 50, 1335–1341. doi:10.1038/s41588-018-0184-y.
